# *Basella alba* L. (Malabar Spinach) as an Abundant Source of Betacyanins: Identification, Stability, and Bioactivity Studies on Natural and Processed Fruit Pigments

**DOI:** 10.1021/acs.jafc.3c06225

**Published:** 2024-02-01

**Authors:** Katarzyna Sutor-Świeży, Renata Górska, Agnieszka Kumorkiewicz-Jamro, Ewa Dziedzic, Monika Bieniasz, Przemysław Mielczarek, Łukasz Popenda, Karol Pasternak, Małgorzata Tyszka-Czochara, Monika Baj-Krzyworzeka, Monika Stefańska, Przemysław Błyszczuk, Sławomir Wybraniec

**Affiliations:** † Department C-1, Faculty of Chemical Engineering and Technology, Cracow University of Technology, ul. Warszawska 24, Krakow 31-155, Poland; ‡ South Australian Health and Medical Research Institute, Adelaide 5000, SA, Australia; § Faculty of Health and Medical Sciences, University of Adelaide, Adelaide 5000, SA, Australia; ∥ Faculty of Biotechnology and Horticulture, University of Agriculture in Krakow, al. 29 Listopada 54, Krakow 31-425, Poland; ⊥ Department of Analytical Chemistry and Biochemistry, Faculty of Materials Science and Ceramics, AGH University of Science and Technology, al. Adama Mickiewicza 30, Krakow 30-059, Poland; # Laboratory of Proteomics and Mass Spectrometry, Maj Institute of Pharmacology, Polish Academy of Sciences, ul. Smętna 12, Krakow 31-343, Poland; ∇ NanoBioMedical Centre, Adam Mickiewicz University, ul. Wszechnicy Piastowskiej 3, Poznan 61-614, Poland; ○ Institute of Bioorganic Chemistry, Polish Academy of Sciences, ul. Noskowskiego 12/14, Poznan 61-704, Poland; ◆ Faculty of Pharmacy, Jagiellonian University Medical College, ul. Medyczna 9, Krakow 30-688, Poland; ¶ Faculty of Medicine, Department of Clinical Immunology, Institute of Pediatrics, Jagiellonian University Medical College, Kraków 30-688, Poland

**Keywords:** decarboxylated and dehydrogenated gomphrenins, betacyanins, betalains, Basella alba, plant pigments

## Abstract

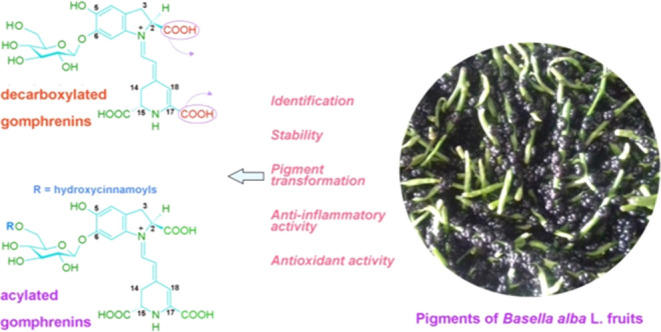

The antioxidant and anti-inflammatory activities of acylated and decarboxylated gomphrenins, as well as *Basella alba* L. fruit extract, were investigated in relation to gomphrenin, known for its high biological potential. The most abundant natural acylated gomphrenins, namely, 6′-*O*-*E*-caffeoyl-gomphrenin (malabarin) and 6′-*O*-*E*-4-coumaroyl-gomphrenin (globosin), were isolated from *B. alba* extract for the studies. In addition, controlled thermal decarboxylation of gomphrenin in the purified *B. alba* extract at 65–75 °C resulted in the formation of the most prevalent decarboxylated products, including 17-decarboxy-gomphrenin and 2,17-bidecarboxy-gomphrenin, along with their isoforms. The structures of the decarboxylated pigments were confirmed by NMR analyses. Exploring the matrix effect on pigment reactivity revealed a tremendous increase in the stability of all betacyanins after the initial stage of extract purification using a cation exchanger under various conditions. This indicates the removal of a substantial portion of the unfavorable matrix from the extract, which presumably contains reactive species that could otherwise degrade the pigments. Furthermore, the high concentration of citrates played a significant role in favoring the formation of 2-decarboxy-gomphrenin to a considerable extent. *In vitro* screening experiments revealed that the tested compounds demonstrated strong anti-inflammatory properties in lipopolysaccharide (LPS)-activated human macrophages. This effect encompassed the selective inhibition of cytokine and chemokine release from activated macrophages, modulation of the chemotactic activity of immune cells, and the regulation of tissue remodeling mediators’ release.

## 1 Introduction

Natural pigments known as betalains (including betaxanthins and betacyanins) have been associated with a range of beneficial health-promoting attributes, including antioxidant, anticancer, and anti-inflammatory properties.^1,2^ These compounds possess vivid coloration^3^ (maintained across a broader pH range compared to anthocyanins),^4^ making them attractive candidates for potential application as natural dyes in food and cosmetic industries.^5,6^ Furthermore, their nonallergenic and nontoxic nature in humans adds to their appeal, positioning them as potential active ingredients in supplements, nutraceuticals, and even pharmaceuticals.^7^

Betalains are valuable food constituents as they are potent radical scavengers. Research has demonstrated that betacyanins extracted from red beet exhibit 1.5–2.0 times greater free-radical scavenging activity compared to anthocyanins such as cyanidin-3-*O*-glucoside and cyanidin at pH above 4.^8,9^ Additionally, certain betacyanin pigments have been confirmed to possess higher antioxidant activity than various natural antioxidants, including β-carotene,^10^ ascorbic acid,^11^ rutin,^12^ catechin,^13^ and α-tocopherol.^14,15^

Betacyanins encompass chemical entities from the gomphrenin group, which can be found in plants of the *Basellaceae* family, particularly in *Basella alba* L. (Figure S1) and its variety *B. alba* L. var. “Rubra” (Malabar spinach).^16^ Plants classified under the Basella genus are edible succulents characterized by small, dark blue stone fruits and distinctive branched, climbing stems with alternate leaves.^17^ These plants are primarily cultivated in subtropical regions, where their potent health benefits have been acknowledged and well-regarded.^18^ Their anti-inflammatory and antibacterial properties are highlighted,^19^ but studies have also reported the cytotoxic effect of Malabar spinach fruit extracts on human cervical carcinoma cells.^20^ Besides containing compounds such as carbohydrates, proteins, lipids, niacin, ascorbic acid, and tocopherols, Malabar spinach is also a rich source of betalain pigments. The dominant pigment present in *B. alba* is gomphrenin (betanidin 6-*O*-β-d-glucoside).^21^

The chemical structures of gomphrenin-based pigments are characterized by a phenolic moiety linked to carbon C-5 and a glucosyl group attached at the C-6 position.^22^ Their strongest antioxidant properties among the basic betacyanins were indicated,^11,23^ but there is not much research on anti-inflammatory^24^ and anticancer^20^ properties reported. It has also been indicated that betacyanin-containing extracts may affect permeability across microbial cell membranes, leading to structural and functional changes and ultimately cell death.^25,26^ In addition to betanin and gomphrenin, numerous acylated betacyanins with unknown properties were reported.^15,27−32^

Low stability is still an important factor hampering betacyanin’s more widespread use. The direction of betacyanin decomposition depends on many factors but above all on the temperature, pH, and the presence of stabilizing factors.^33^ These are, among others, ascorbic acid,^34^ isoascorbic acid,^35−37^ or chelating agents such as citric acid and ethylenediaminetetraacetic acid (EDTA).^36,38^ By appropriately optimizing processing parameters such as temperature, pH, and concentration of stabilizing additives, it becomes possible to control the decomposition pathways of betacyanins.^33^ In this report, the influence of citric acid and EDTA on the stability and reactivity of gomphrenin and its acylated derivatives during heating processes was studied for nonpurified *B. alba* extract and chromatographically purified extracts. For this aim, *B. alba* plants were cultivated in a greenhouse within a temperate climate (Cracow, Poland), with the goal of obtaining fruit extracts for studies. This enabled the determination of the impact of the complex *B. alba* fruit matrix on degradation of the pigments as well as pathways of decarboxylated pigments’ formation. Based on liquid chromatography–mass spectrometry (LC–MS) and NMR pigment identification, a method for the generation of decarboxylated gomphrenins for further bioactivity studies was developed.

In the human body, macrophages are the first line of defense against pathogens that invade tissues. Innate immune cells play a vital role in employing numerous defense mechanisms against invading pathogens, effectively preventing infections. In particular, they secrete cytokines and chemokines that help coordinate the innate and adaptive immune responses of the body. Macrophages are classically activated by an antigen of the bacterial cell wall, lipopolysaccharide (LPS) acting via Toll-like receptor 4 (TLR-4) pathway to synthesize cytokines such as tumor necrosis factor α (TNFα), interleukin-1β (IL-1β) and interleukin-6 (IL-6), the main proinflammatory cytokines with systemic effects.^39^ However, these regulatory molecules not only exhibit proinflammatory properties but also modify the vascular endothelial function, facilitate leukocyte migration into the inflammatory sites and exerts chemotactic effects on immune cells. Therefore, macrophages also play a crucial role in physiological processes such as wound healing.^40^

A growing body of evidence indicates that betalains display potent anti-inflammatory properties, which was demonstrated *in vitro*,^41^*in vivo*,^42^ and in clinical trials.^43^ Hence, we used an xMAP-based multiplex immunoassay^44^ to determine whether gomphrenin-type betacyanins and *B. alba* extract mitigates inflammation in LPS-activated human macrophages. The impact of gomphrenin derivatives on the mechanisms underlying acute and chronic inflammation has not been fully characterized yet, though some preliminary results have been reported.^45−47^

With respect to gomphrenin-based betacyanins, the antioxidant properties and bioactivity of the majority of compounds remain untested. Therefore, in the subsequent discussion, we also emphasize the examination of the properties of purified and isolated compounds extracted from *B. alba* fruits. This includes selected acylated compounds as well as derivatives obtained through thermal decarboxylation.

## 2 Experimental Section

### 2.1 Reagents

Formic acid, acetone, LC–MS grade methanol, and water were obtained from Sigma Chemical Co. (St. Louis, MO). Reagents for antioxidant activity assays, trolox (6-hydroxy-2,5,7,8-tetramethylchroman-2-carboxylic acid), AAPH (2,2′-azo-bis(2-amidinopropane)-dihydrochloride), sodium fluorescein, TPTZ (2,3,5-triphenyltetrazolium chloride), ferric chloride hexahydrate, sodium acetate trihydrate, ABTS (2,2′-azino-bis(3-ethylbenzothiazoline-6-sulfonic acid) diammonium salt), and caffeic acid, were purchased from Sigma-Aldrich (St. Louis, MO).

### 2.2 Plant Material

The seeds of *B. alba* L. obtained from the Botanical Garden of the Jagiellonian University Institute of Botany (Cracow, Poland) were sown in a greenhouse of the University of Agriculture in Cracow (Faculty of Biotechnology and Horticulture). Sowing of the seeds was done in a 3:1 ratio of soil and coconut pith mass and watered daily. The seedlings were transplanted to fertile soil with an increased amount of organic matter and pH 6.5–6.8. The plants were intentionally cultivated to facilitate the climbing behavior of the vines and promote rapid growth. Consequently, they were trellised to attain a height of up to 3 m. The plants were grown at a controlled temperature and moisture for proper flowering and fruiting (Figure S1). Matured *B. alba* fruits were collected from the University of Agriculture in Cracow and designated for further research.

### 2.3 Preparation of *B. alba* Fruit Extracts

*B. alba* fruits (1 kg) were manually squeezed, and the obtained juice was centrifuged before filtering through a 0.2 mm i.d. pore size filter. The filtered juice was then diluted 3-fold with water and stored at −20 °C for preservation over several weeks. Prior to subsequent experiments, the extract was further refined by passing it through a 10 cm height × 2 cm i.d. bed of 0.063/0.200 mm silica (J.T. Baker, Deventer, Holland) to remove hydrocolloids and proteins, resulting in a clear solution. A portion of the obtained *B. alba* extract B1 underwent subsequent purification using open column chromatography on a strongly acidic cation-exchange resin (Strata X-C, Phenomenex, Torrance, CA) to yield the refined extract B2. For this process, the 0.1 M HCl acidified extract B1 was applied to the top of the column (40 mm i.d. × 250 mm height), followed by column rinsing with 0.1 M HCl. The betacyanin fraction was then eluted with water, and the eluates were pooled and concentrated using a rotary evaporator under reduced pressure at 25 °C to obtain the extract B2.

Extract B2 was further purified by flash chromatography on a silica C18 sorbent (Chromabond, Macherey-Nagel, Germany) in a column of 35 mm i.d. × 130 mm height, resulting in the final extract B3. The procedure involved applying the aqueous extract to the top of a methanol-activated and water-conditioned column, followed by column rinsing with water. The betacyanin fraction was eluted using an eluent composed of water/methanol/formic acid, 48/50/2 (v/v/v). The pigment fraction was then concentrated using a rotary evaporator under reduced pressure, resulting in the extract B3.

Most of the neutral and positively charged compounds can pass through the cationic exchanger during obtaining extract B2. This is well-known that one of the groups are sugars. They are also removed in the second cleanup stage on ODS columns during obtaining extract B3; however, on ODS columns, the separation of the pigments is more fine because it processes according to polarity. Therefore, much more polar compounds are eluted much faster than the pigments from the columns, as well as the more hydrophobic compounds, such as flavonoids, stay adsorbed onto the stationary ODS phases.

### 2.4 Experiments on the Influence of Citrates and EDTA on *B. alba* Fruit Extract Stability

An investigation on the influence of citrates and EDTA on the stability of heated *B. alba* fruit extracts B1, B2, and B3 as well as the generation of various gomphrenin derivatives was performed. This was carried out following a previously published procedure, with modifications introduced due to the inclusion of the reagents.^48^ The aqueous stock solutions of the extract containing betacyanins, with a concentration of 600 μM expressed in terms of gomphrenin equivalents, were appropriately diluted in microplate wells to obtain tested solutions at concentrations of 15, 30, and 60 μM. Each well contained 20 μL of acetate/phosphate or citrate buffers at pH 3–8 (20 mM), and selected wells also contained 20 μL of 0.2 mM EDTA solution, all brought up to a final volume of 200 μL. These samples were heated at 30, 50, or 85 °C in a thermostat for 72 h, 8, or 1 h, respectively, and monitored spectrophotometrically in a microplate reader Tecan Infinite 200 (Tecan Austria GmbH, Grödig/Salzburg, Austria). During the experiments, additional aliquots (20 μL) of the heated samples were taken for LC-DAD-ESI-MS/MS analyses after 10x dilution. All of the experiments were performed in triplicate.

To optimize the generation of 2- and 2,17-decarboxy-gomphrenin, we conducted analogous experiments at 65, 70, and 75 °C. Notably, a higher concentration of citric acid (100 mM) was utilized in these studies.

### 2.5 Formation of Gomphrenin Derivatives in Semipreparative Scale for Bioactivity Assays

Thermal decarboxylation of gomphrenin at specific positions was carried out in a diluted 2 L *B. alba* fruit extract B2 solution (total betacyanin concentration of 30–60 μM), guided by the outcomes of the heating experiments detailed in Section 3.3. Pigment 17-decarboxy-gomphrenin was generated in a 60 μM extract B2 aqueous solution containing 100 mM citric acid, following a 3 h heating at 65 °C. Similarly, the pigments 2-decarboxy- and 2,17-bidecarboxy-gomphrenin were generated in a 30 μM extract B2 aqueous solution with 100 mM citric acid, subjected to 2–3 h of heating at 70 °C. The resulting solutions were adsorbed onto a silica C18 (Chromabond) column, and the pigments were subsequently eluted and concentrated as outlined in the previous section. Finally, the pigments were separated through preparative high-performance liquid chromatography (HPLC).

### 2.6 Isolation and Purification of Betacyanins from Extracts

To identify gomphrenin-type pigments intended for the bioactivity studies, betacyanins extracted from *B. alba* extract B3 were purified using preparative high-performance liquid chromatography (prep-HPLC). Similarly, decarboxylated gomphrenin fractions, previously generated through thermal decarboxylation of gomphrenin (as detailed in Section 3.3), were also isolated and purified. These fractions had undergone preliminary purification via flash chromatography on a silica C18 sorbent.

The concentrated gomphrenin derivatives were subsequently separated using a Shimadzu LC-20AD system, employing an HPLC semipreparative column Synergy Hydro-RP 250 mm × 30 mm i.d., 10 μm (Phenomenex), along with a 20 mm × 25 mm i.d. guard column of the same material (Phenomenex). A typical gradient system consisting of 0.5% aqueous formic acid (solvent A) and acetone (solvent B) was used as follows: 0 min, 16% B; increasing to 8 min, 18% B; increasing to 15 min, 22% B; increasing to 25 min, 26% B; increasing to 35 min; 82% B. The injection volume was 25 mL with a flow rate of 35 mL/min. Detection was performed using a UV–vis detector at 540 and 510 nm at a column temperature of 22 °C. The eluates were pooled and concentrated in a rotary evaporator at 25 °C under reduced pressure to remove the organic solvent and stored at −20 °C for further studies.

### 2.7 Chromatographic Analysis with Detection by a Low-Resolution Mass Spectrometric System (LC-DAD-ESI-MS/MS)

For the chromatographic and low-resolution mass spectrometric (LRMS) analyses, a mass spectrometric system (model LCMS-8030, Shimadzu, Kyoto, Japan) coupled to LC-20ADXR HPLC pumps, an injector model SIL-20ACXR, and a PDA detector (photodiode array) model SPD-M20A, all controlled with LabSolutions software version 5.60 SP1 (Shimadzu, Japan), was used. The samples were eluted through a 150 mm × 4.6 mm i.d., 5.0 μm, Kinetex C18 chromatographic column preceded by a guard column of the same material (Phenomenex, Torrance, CA). The injection volume was 20 μL, and the flow rate was 0.5 mL/min. The column was thermostated at 40 °C. The separation of the analytes was performed with binary gradient elution. The mobile phases were A, 2% formic acid in water and B, methanol. The gradient profile was (*t* (min), % B), (0, 10), (12, 40), (15, 80), (19, 80). The full-range PDA signal was recorded, and individual chromatograms were displayed at 538, 505, 490, and 440 nm. Positive ion electrospray mass spectra were recorded using the LC–MS system, controlled by LabSolutions software. The ionization electrospray source operated in positive mode (ESI+), with an electrospray voltage of 4.5 kV and a capillary temperature of 250 °C. N_2_ gas was used for the spray. The system recorded total ion chromatograms, mass spectra, and ion chromatograms in the selected ion monitoring mode (SIM), as well as the fragmentation spectra. Argon was used as the collision gas for the collision-induced dissociation (CID) experiments. The relative collision energies for MS/MS analyses were set at −35 V on an arbitrary scale.

### 2.8 Chromatographic Analysis with Detection by a High-Resolution Mass Spectrometric System (LC-Q-Orbitrap-MS)

All high-resolution mass spectra were analyzed using an Orbitrap Exploris 240 Mass Spectrometer with Xcalibur software version 4.5.445.18 (Thermo Fisher Scientific, Brema, Germany) coupled to an HPLC Dionex UltiMate 3000 chromatographic separation system. The chromatographic conditions were the same as for the LRMS experiments.

The conditions for positive thermally focused/heated electrospray (HESI) were as follows: capillary voltage, 3.5 kV; capillary temperature, 250 °C; the sheath gas, auxiliary gas, and sweep gas flow rate were set at 50, 15, and 3 arbitrary units, respectively; probe heater temperature, 350 °C; S-lens RF level, 55%. The full-scan selection of target betacyanins in the LRMS system was conducted in a positive polarity mode. The MS data were acquired in the *m*/*z* 300–1100 range with a resolution (full width at half-maximum, fwhm, at *m*/*z* 200) of 120,000. The automatic gain control (AGC) target value was 240,000 in the full-scan mode. The maximum isolation time was set to auto mode.

Product ion scan mode was used as the acquisition mode, where targeted precursors were isolated and fragmented in the high-energy collision dissociation (HCD) cell. Selected precursor ions were fragmented in the higher-energy collisional activated dissociation cell, and the fragment (MS2) ions were analyzed in the Orbitrap analyzer. During the MS^2^ experiments for selected target betacyanins, generated fragmentation ions were collected in the high-energy collision dissociation (HCD) mode at collision energies of 30 and 50 eV. The automatic gain control (AGC) target value and the resolution were 45,000 and 30,000, respectively. The *m*/*z* range was 80–800, and the maximum isolation time was set to 60 ms. The number of microscans per MS/MS scan was set to 1 and the isolation window to *m*/*z* of 1.5.

### 2.9 NMR Experiments

The NMR data were acquired on a Bruker Avance III 700 spectrometer (Bruker Corp., Billerica, MA) using a QCI CryoProbe at 295 K in nonacidified D_2_O (for 2-dGp) and CD_3_OD acidified by trifluoroacetic acid-d (for 17-dGp and 2,17-dGp). All one-dimensional (1D) (^1^H, ^13^C) and two-dimensional (2D) [COSY, HSQC, HMBC, TOCSY, and NOESY (gradient-enhanced)] experiments were performed using standard pulse sequences and acquisition parameters.^33^ The residual water peak for the measurements carried out in D_2_O was suppressed using the low-power presaturation. Chemical shifts were referred to internal 3-(trimethylsilyl)-2,2,3,3-tetradeuteropropionic acid (TMSP-*d*_4_) (δ_H_ = 0.00 ppm, δ_C_ = 0.0 ppm) or residual CD_3_OD (δ_H_ = 3.31 ppm, δ_C_ = 49.0 ppm).

### 2.10 Antioxidant Activity Measurements

ABTS, FRAP, and ORAC assays were performed to evaluate the antioxidant activity of *B. alba* extracts B1 and B2, as well as the isolated pigments. The tested samples were prepared by diluting 1 mg of lyophilizate in 1 mL of distilled water. UV–vis spectrophotometric and fluorescence measurements were conducted using a Tecan Infinite 200 microplate reader (Tecan Austria GmbH, Grödig/Salzburg, Austria). Each measurement for every sample was carried out in triplicate across three independent experiments.

#### 2.10.1 ABTS Assay

A solution of cation radicals (ABTS^+•^) was prepared by reacting a 3.5 mM aqueous solution of potassium persulfate (K_2_S_2_O_8_) with a 2 mM solution of 2,2′-azino-bis(3-ethylbenzothiazoline-6-sulfonic acid) (ABTS), which was then diluted with water to achieve a final ratio of 1:0.35:0.65 ABTS:K_2_S_2_O_8_:H_2_O (v/v/v). This prepared solution was incubated at room temperature in a light-protected environment for 8 h.

A solution of trolox (1 mM) was prepared by dissolving 2.5 mg of trolox in 10 mL of ethanol. The resulting solution was then diluted 5-fold for use as a standard.

Increasing amounts of each sample and trolox were applied to a 96-well plate, followed by the addition of 30 μL of aqueous ABTS^+•^ solution with an initial absorption of 1. The total volume of the reaction solution was 200 μL. Sample concentrations were adjusted to reduce the absorbance of ABTS cation radicals within the 10–90% range compared to the control.

After preparation, the samples were incubated in darkness at room temperature for 30 min. Subsequently, they were shaken for 20 s, and spectrophotometric measurements were performed at 734 nm. The results were expressed as IC_50_ values and in mmol trolox/g DW (millimoles of trolox per gram of dry weight of the lyophilized sample).

#### 2.10.2 Frap Assay

The method described by Benzie and Strain^49^ was followed. To prepare the FRAP reagent, a mixture of 10 mM TPTZ solution in 40 mM aqueous HCl, a 20 mM FeCl_3_ solution, and a 300 mM sodium acetate buffer (pH 3.6) was combined in a ratio of 1:1:10 (v/v/v). The FRAP assay was performed by mixing 100 μL of the reagent with tested samples and water, resulting in a final volume of 200 μL. The prepared solutions were incubated for 10 min at room temperature, and then the absorbance was measured at 593 nm. The antioxidant activity was compared to the positive control, a standard trolox solution. The results were expressed as mmol trolox/g.

#### 2.10.3 ORAC Assay

For the ORAC assays, 150 μL of 23 nM fluorescein solution, 25 μL of 75 mM sodium phosphate buffer (pH 7.4), and 25 μL of a test sample solution were added to each well of a 96-well microplate and incubated for 30 min at 37 °C. A trolox solution (0–50 μM) was used as the standard. After a 30 min incubation, 25 μL of 153 mM 2,2′-azo-bis(2-amidinopropane)dihydrochloride (AAPH) solution was introduced into the wells to initiate the reaction. The plate was shaken for 10 s. The reaction kinetics were determined through fluorescence measurements (excitation at 485 nm, emission at 528 nm), taken every minute for 1 h. The antioxidant activity was calculated based on the AUC (area under curve) and the net AUC (net area under the curve) of both the standards and samples. The results were expressed as mmol trolox/g DW.

### 2.11 Cell Isolation and Culture

Anticoagulated citrate dextrose-A-treated blood from healthy donors was purchased from the Regional Center of Blood Donation and Blood Therapy in Cracow, Poland (agreement no. DZM/SAN/CM/U-678/2015 RCKiK/CMUJ). Peripheral blood mononuclear cells (PBMCs) were isolated by centrifugation using the standard Pancoll density gradient (Panbiotech, Aidenbach, Germany). Monocytes were separated from PBMCs as described previously^50^ by countercurrent centrifugal elutriation (JE-6B elutriation system) equipped with a 5 mL Sanderson separation chamber (Beckmann-Coulter, Palo Alto, CA). Cells were washed with Dulbecco’s phosphate buffered saline (PBS) (Gibco, NY), resuspended in RPMI 1640 medium (Gibco), and kept in an ice bath until used. Then, the purity of isolated human monocytes was confirmed by flow cytometry using an FACSCanto flow cytometer (BD Biosciences Immunocytometry Systems, San Jose, CA) using anti-CD14 antibody (BD Biosciences Pharmingen, San Diego, CA). Isolated monocytes were then cultured in ultralow attachment 24-well plates (Corning Incorporated, Corning, NY) at a density of 1 × 10^6^ cells/mL per well and cultured in RPMI 1640 medium (Gibco) supplemented with 2 mM l-glutamine, 10% ultralow endotoxin fetal bovine serum (Biowest, France), and 100 U/mL penicillin/100 μg/mL streptomycin mixture (Gibco). The cells were incubated at 37 °C, 5% CO_2_. Medium was exchanged with a fresh one every 2 days. After 7 days of passaging, human monocytes spontaneously differentiated and exhibited morphological and phenotypical characteristics of macrophages.

For experiments, human monocyte-derived macrophages were treated with *B. alba* extract B2 or isolated pigments (20 μM) for 24 h and subsequently incubated for the next 24 h with 0.1 μg/mL of lipopolysaccharide (LPS) from *Salmonella abortus* equi S-form (Enzo Life Sciences) and appropriate extracts. Then, the solutions were aspirated and analyzed by the immunoassay tests (Section 2.13).

Control cells (without tested samples nor LPS) were incubated in the medium only with a 10% addition of purified water (Milli-Q Ultrapure Water Systems), solvent to gomphrenins. Positive controls (cells exposed to LPS for 24 h with the addition of water and without tested compounds) were included to exclude the influence of nonspecific factors on macrophage activation.^51^ The screening of tested samples for endotoxin contamination was performed using Pierce Chromogenic Endotoxin Quant Kit (Thermo Scientific).

### 2.12 Cell Viability Assay

3-(4,5-Dimethylthiazol-2yl)-2,5-diphenyl tetrazolium bromide (MTT) colorimetric assay is a widely used end-point method that measures the cellular metabolic rate to assess the potential inhibitory effect of chemicals on cells upon treatment. For the assay, human macrophages were pretreated with each tested compound (20 μM) and then exposed to LPS for the next 24 h. 3-(4,5-Dimethylthiazol-2yl)-2,5-diphenyl tetrazolium bromide (MTT), purchased from Sigma-Aldrich assay was performed as described previously.^52^ Doxorubicin (Sigma-Aldrich) was used as a reference (DOX) at a concentration of 100 μL. The absorbance of MTT formazan was recorded at 550 nm (the reference wavelength was 690 nm) using a microplate reader Infinite M200 Pro, Tecan, Austria, and a percentage of viable cells was calculated compared to positive control (100%).^53^

### 2.13 Detection of Inflammatory Biomarkers with xMAP-Based Multiplex Immunoassay

After 24 h of preincubation of human macrophages with tested compounds and the extract, and following incubation with LPS, the cell culture supernatants were collected and then centrifuged (6500*g*, 10 min) to remove debris. The level of inflammation modulatory molecules secreted from macrophages was measured in media using antibody microarray Luminex Human Discovery Assay (Biotechne, France) following the manufacturer’s protocol. Fluorescence intensity was recorded using a Luminex LX-200 flow-based bead reader with software (Bio-Techne, MN). The analysis was performed using an xMAP Luminex system with color-coded microsphere beads coated with biotinylated antibodies specific to cytokines (CCL2/MCP-1, CXCL1/GRO-α, TNF-α, IL-1β/IL-1F2, IL-6, IL-8/CXCL8, IL-18/IL-1F4, MMP-9, and VEGF). Streptavidin–phycoerythrin, which binds to the biotinylated antibodies, was used in analysis. Appropriate standards were prepared according to the protocol. Each supernatant was 2-fold-diluted in Calibrator Diluent RD6-52 before the assay.

### 2.14 Statistical Analysis

All experiments were conducted in triplicate. The bioactivity data were analyzed using the commercially available packages Statistica PL v.10 (StatSoft, Tulsa, OK). Statistical significance analysis was performed by one-way analysis of variance (ANOVA) followed by the Tukey post hoc test. The levels of *p* < 0.05 were considered as statistically significant. All data presented in figures were expressed as arithmetic mean values and standard deviation (SD).

## 3 Results and Discussion

The natural pigments, including gomphrenin and its acylated derivatives, are present at high concentrations in *B. alba* L. fruits, serving as an alternative rich source of betacyanins. LC-DAD-MS fingerprints of the natural acylated gomphrenins were presented in the recent study.^21^ The total concentration of betacyanins can be expressed in gomphrenin or betanin equivalents, which was determined for *B. alba* mature fruits to be 42.0 mg/100 g.^21^

The fractions of gomphrenin/isogomphrenin and all acylated betacyanins constitute 53.4 and 38.6%, respectively. Among the acylated pigments, 6′-*O*-*E*-4-coumaroyl-gomphrenin/isogomphrenins (globosin/isoglobosin) were found to be the most abundant, comprising approximately 14.7% of the fraction. Another acylated pigment, 6′-*O*-*E*-caffeoyl-gomphrenin/isogomphrenin (malabarin/isomalabarin), was also present but in a lower proportion, accounting for around 2.8%.^21^

Research on *B. alba* fruit extracts and their processed products has the potential to significantly contribute to our understanding of the bioactivity of gomphrenins. The thermal processing of *B. alba* extracts leads to the presence of decarboxylated derivatives of gomphrenin, which could influence their prohealth properties. Furthermore, this discovery might broaden the scope of their potential applications in the food industry.

**Figure 1:**
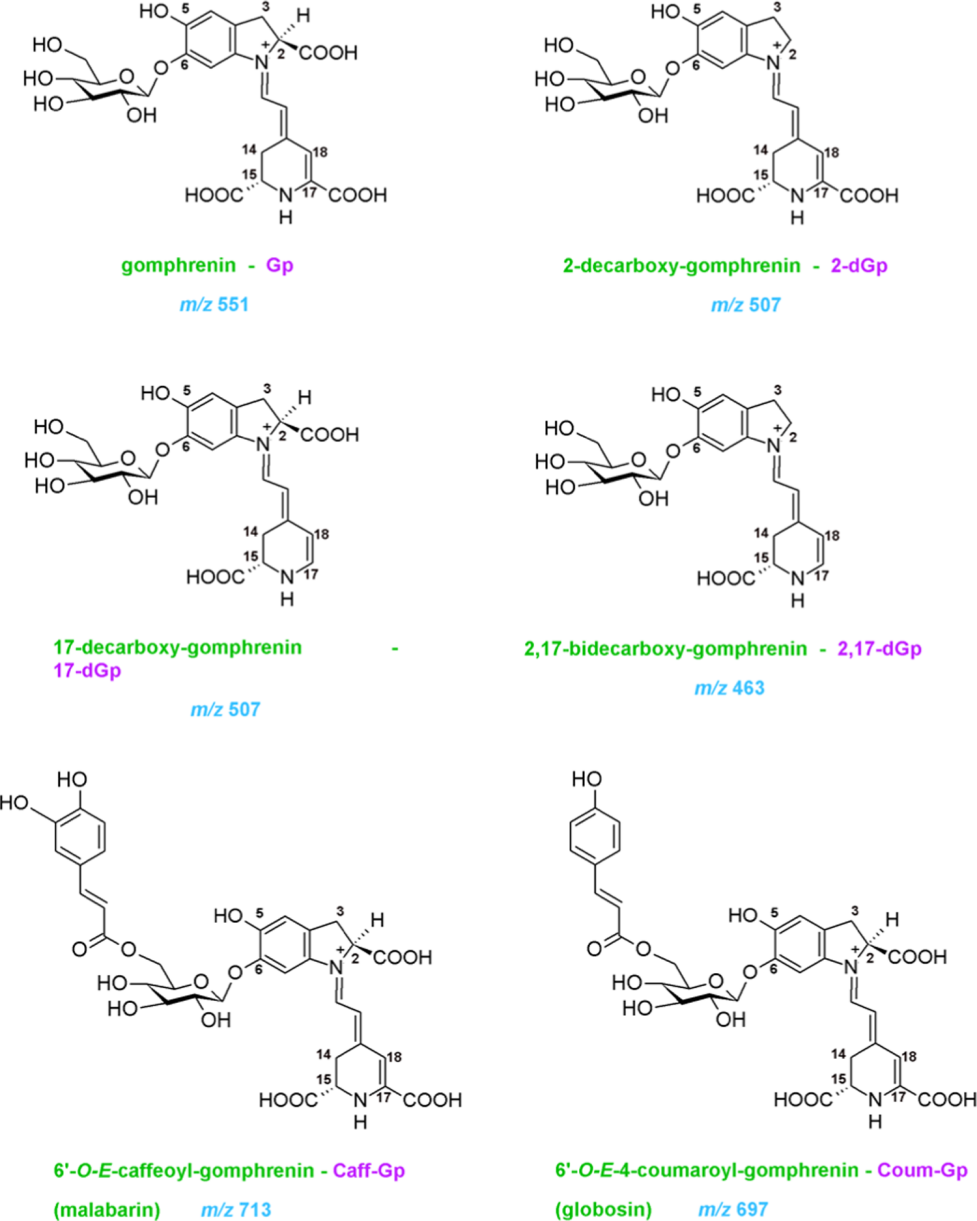
Chemical structures of gomphrenin and its decarboxylated and acylated derivatives dedicated to biological assays.

Figure 1 depicts the chemical structures of the pigments subjected to the bioactivity assays. The investigated pigments include gomphrenin, both acylated and decarboxylated gomphrenins, along with *B. alba* fruit extracts. Gomphrenin was selected as the reference pigment being previously identified as the most antioxidative betacyanin.^11^ Additionally, the naturally occurring acylated gomphrenins, namely, globosin and malabarin, were selected for bioactivity assessment.

A tentative identification of nonacylated gomphrenin derivatives (mono-, bi-, and tridecarboxylated gomphrenins) generated during the heating of gomphrenin in diluted *B. alba* fruit juice was previously reported,^54^ along with their chromatographic profiles. Furthermore, the chemical structures of gomphrenin and acylated derivatives, namely, malabarin and globosin, were fully elucidated in a prior study.^55^ To further expand the group of newly characterized gomphrenin pigments, in this contribution, we confirmed the chemical structures of decarboxylated derivatives such as 2-decarboxy-gomphrenin, 17-decarboxy-gomphrenin, and 2,17-bidecarboxy-gomphrenin for the first time using the NMR method. All the gomphrenin derivatives were designated for evaluation of their antioxidant and anti-inflammatory properties.

To enhance the stability of *B. alba* extracts and purified gomphrenin pigments, we conducted experiments to assess the thermal stability of gomphrenin and its acylated derivatives in both purified (B2 and B3) and nonpurified (B1) *B. alba* extracts. Additionally, novel decarboxylated and dehydrogenated acylated gomphrenins were tentatively identified by LC–MS. Furthermore, we investigated the influence of citric acid and EDTA, known stabilizing agents in the food industry, on the formation of acylated derivatives during the heating experiments.

Based on the results obtained, experiments on the selective generation of 2-decarboxy-gomphrenin, 17-decarboxy-gomphrenin, and 2,17-bidecarboxy-gomphrenin under the influence of an increased concentration of citric acid (100 mM) were performed. This enabled us to obtain preparative quantities of the pigments for bioactivity studies.

### 3.1 Novel Derivatives of Acylated Gomphrenins Generated by Heating

Heating experiments were conducted on isolated natural acylated gomphrenins from *B. alba* fruits, namely, *p*-coumaroylated gomphrenin (globosin) **13**, caffeoylated-gomphrenin (malabarin) **21**, feruloylated gomphrenin (basellin) **29**, and sinapoylated gomphrenin (gandolin) **37**. The chemical structures of these compounds (Figure S2) have recently been confirmed by NMR.^21^

These experiments revealed the generation of novel decarboxylated and dehydrogenated derivatives at 85 °C, which were tentatively identified by LRMS. While the LC–MS system provided *m*/*z* values for the new pigments, the UV–vis data obtained through PDA detection unequivocally indicated the positions of decarboxylation in the isomeric monodecarboxylated pigments. This determination was based on previous findings obtained for nonacylated betanins and gomphrenins,^30,31,54^ and it further supported the identification of oxidized derivatives.

**Figure 2:**
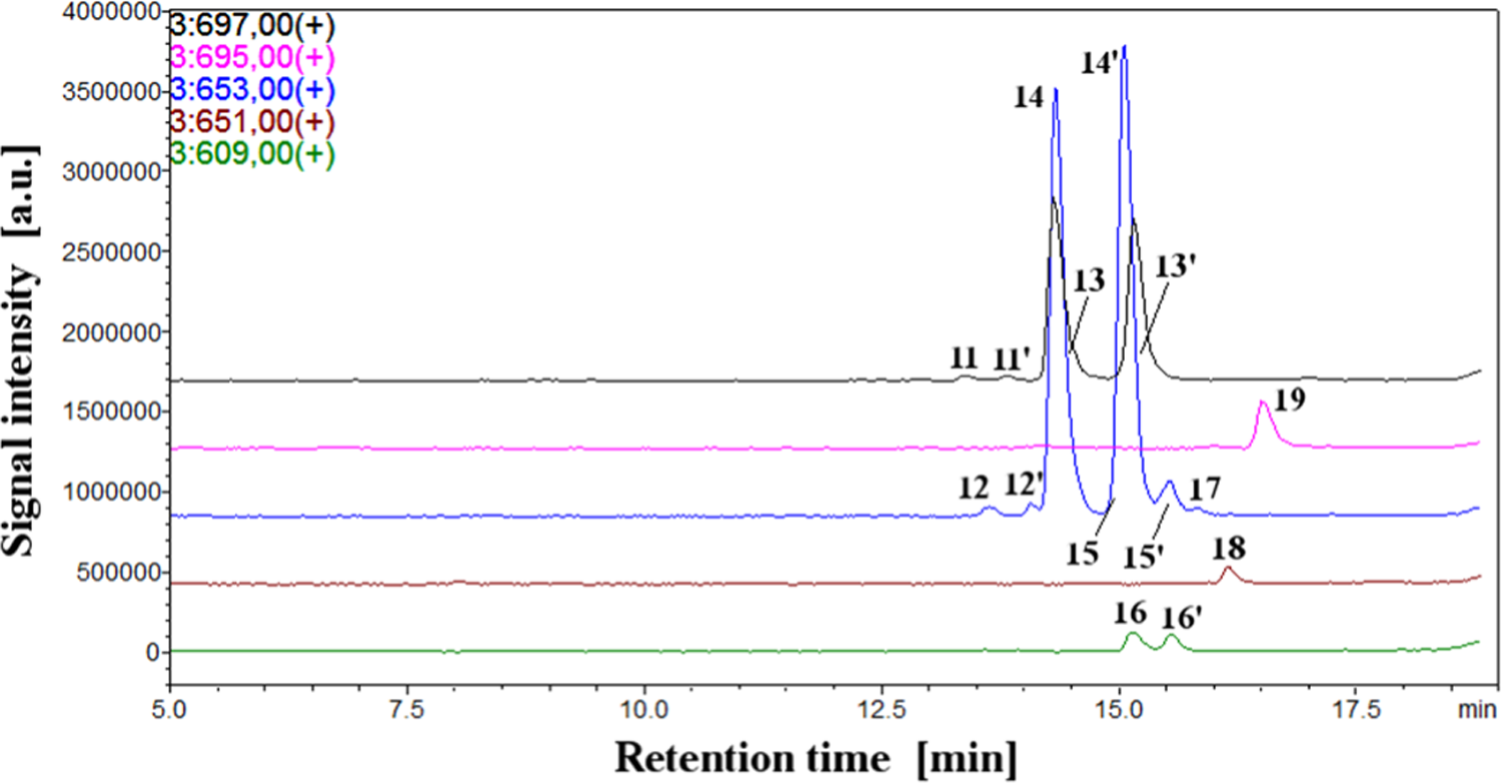
Chromatograms of selected ions monitored in the LC–MS system for globosin and its derivatives generated during heating experiments.

Globosin **13** and its generated derivatives **11**–**12** and **14**–**19** (Table 1) were separated using a C18 HPLC column and subsequently detected by the PDA-MS system (Figure 2). Their elution profiles differed from analogous nonacylated gomphrenin derivatives **2**–**10** (Table S1 and Figure S3).^30,31,54^ Notably, the most significant distinctions were observed in relation to the faster elution of 17-decarboxy-isoglobosin **12′** from its precursor, globosin **13′**, as well as lower retention time for 2-decarboxy-globosin **15** compared to 17-decarboxy-isoglobosin **12′** (Figure 2). These deviations significantly contrasted with the typical behavior observed in betacyanins, as observed in previous research findings.^30,31,54^ Similar elution orders were also determined for the derivatives of isolated malabarin **21**, basellin **29**, and gandolin **37** (Tables S2–S4).

**Table 1:** Chromatographic, Spectrophotometric, and Mass Spectrometric Data of the Analyzed Globosin-Based Betacyanins Present in *B. alba* Fruit Extracts B1, B2, and B3 Submitted to Heating at 85 °C

no.	compound	abbreviation	*R*_t_ [min]	λ_max_ [nm]	*m*/*z*	*m*/*z* MS/MS of [M + H]^+^
**11**	*cis*-globosin	cisGb	13.4	544	697	551; 389
**12**	17-decarboxy-*cis*-globosin^a^	17-cisdGb	13.6	511	653	507; 345
**11′**	*cis*-isoglobosin	cisIGb	13.9	544	697	551; 389
**12′**	17-decarboxy-*cis-*isoglobosin^a^	17-cisdIGb	14.1	511	653	507; 345
**13**	globosin	Gb/Coum-Gp	14.3	544	697	551; 389
**14**	17-decarboxy-globosin^a^	17-dGb	14.4	511	653	507; 345
**15**	2-decarboxy-globosin^a^	2-dGb	14.9	538	653	507; 345
**14′**	17-decarboxy-isoglobosin^a^	17-dIGb	15.1	511	653	507; 345
**13′**	isoglobosin	IGb/Coum-IGp	15.2	544	697	551; 389
**16**	2,17-bidecarboxy-globosin^a^	2.17-dGb	15.2	513	609	463; 301
**15′**	2-decarboxy-isoglobosin^a^	2-dIGb	15.5	538	653	507; 345
**16′**	2,17-bidecarboxy-isoglobosin^a^	2.17-dIGb	15.6	513	609	463; 301
**17**	15-decarboxy-globosin^a^	15-dGb	15.8	532	653	507; 345
**18**	2-decarboxy-xanglobosin^a^	2-dXGb	16.2	459	651	505; 343
**19**	neoglobosin^a^	NGb	16.5	484	695	549; 387

a Tentatively identified.

During the stability and reactivity studies (Section 3.2), the globosin derivatives demonstrated the highest abundance due to their prevalent presence in the tested *B. alba* extracts. As a result, the reactions of this pigment were conveniently studied in greater detail. The *cis* configurational isomers **11/11′** of globosin also underwent the decarboxylation reaction, and the most abundant 17-decarboxy-*cis*-globosin/-isoglobosin **12/12′** were observed as the earlier eluted peaks (Figure 2).

HRMS analyses of the **12**, **15**, and **17** structures yielded the isomeric protonated molecular ions at *m*/*z* similar to predicted 653.1977 and their fragmentation (Table S5), resulting in the initial detachment of CO_2_ (653 – 44 = 609 Da) and coumaroyl moiety (653 – 146 = 507 Da) as well as the subsequent generation of deglucosylated (507 – 162 = 345 Da), decarboxylated (345 – 44 = 301; 301 – 44 = 257 Da), and dehydrogenated (257 – 2 = 255 Da) fragments. Determination of retention times and absorption maxima λ_max_ 511, 534, and 530 nm^30,31,54^ enabled the precise assignation of chromatographic peaks to 17-decarboxy-globosin/isoglobosin **12/12′** and 2-decarboxy-globosin/isoglobosin **15/15′** as well as much less abundant 15-decarboxy-globosin **17**.

The detection of 2,17-bidecarboxy-globosin/isoglobosin **16/16′** [with *m*/*z* values closely matching the predicted 609.2079 Da (Table S5)], resulting from the double decarboxylation of globosin/isoglobosin, was supported by their retention times and absorption maxima λ_max_ of 514 (Table 2). The HRMS fragmentation experiments (Table S5) performed on protonated molecular ions yielded decoumaroylated ions (609 – 146 = 463 Da), which underwent subsequent processes including deglucosylation (463 – 162 = 301 Da), decarboxylation (301 – 44 = 257 Da), and dehydrogenation (257 – 2 = 255 Da).

**Table 2:** NMR Data Obtained for the Novel Decarboxylated Gomphrenins, Isolated from *B. alba* L. Heated Extracts^a^

	17-decarboxy-gomphrenin (**2**)		2-decarboxy-gomphrenin (**4**)		2,17-bidecarboxy-gomphrenin (**5**)	
	CD_3_OD/d-TFA		D_2_O		CD_3_OD/d-TFA	
no.	^1^H^b^	^13^C^c^^,^^d^	^1^H^b^	^13^C^c^^,^^d^	^1^H^b^	^13^C^c^^,^^d^
**2** or 2a/b	5.21, *bm*	63.8	3.89, *dd*, 3.2; 10.0	50.9	4.19, *bdd*	51.2
**3a/b**	3.67, *bm*	34.0	3.07, *dd*, 10.2; 16.3	27.9	3.21, *dd*, 10.4; 16.6	27.8
	3.30 (overlap)					
**4**	6.84, *s*	113.7	6.90, *s*	106.6	6.85 (overlap)	113.8
**5**		148.0		140.5		148.1
**6**		147.3		143.0		147.0
**7**	7.51, *s*	102.7	7.25, *s*	100.9	7.53, *s*	102.7
**8**		135.8		132.1		135.6
**9**		127.2		121.9		130.2
**10**		172.2				
**11**	8.31, *d*, 12.5	144.8	7.98, *d*, 12.3	144.3	8.32, *d*, 12.5	144.6
**12**	5.84, *d*, 12.3	105.1	5.83, *d*, 12.2	107.3	5.97, *d*, 12.4	105.8
**13**		163.6		163.0		162.7
**14a/b**	3.38 (overlap)	27.5	3.02, *dd*, 17.5; 5.1	27.6	3.36, *bm*	27.5
	3.33 (overlap)		3.12, *bs*			
**15**	4.55, *bm*	53.3	4.25, *bt*, 7.2	54.7	4.50, *bt*, 9.2	53.2
**17**	7.65	156.1		150.7	7.54	154.4
**18**	5.85, *bs*	105.6	6.13, *bs*	105.4	6.21, *s*	115.5
**19**		172.1		170.1		172.6
**20**				171.4		
**1′**	4.86, *d*, 7.1	104.4	5.01, *d*, 7.7	102.9	4.87, *d*, 6.9	104.3
**2′**	3.49 (overlap)	77.7	3.63 (overlap)	76.4	3.49 (overlap)	77.7
**3′**	3.54 (overlap)	74.9	3.64 (overlap)	73.9	3.54 (overlap)	74.8
**4′**	3.35 (overlap)	71.7	3.51 (overlap)	70.6	3.43 (overlap)	71.6
**5′**	3.56 (overlap)	78.7	3.65 (overlap)	77.1	3.56 (overlap)	78.4
**6′a/b**	4.01, *dd*, 11.9; 4.9	62.8	3.98, *dd*, 11.9; 5.3	61.5	3.97 (overlap)	62.5
	3.69, *dd*, 12.1; 2.2		3.77, *dd*, 11.8; 2.5		3.75 (overlap)	

a The ^1^H and ^13^C NMR spectra of the pigments are presented in Figures S9–S14.

b ^1^H NMR δ [ppm], mult, *J* [Hz].

c ^13^C NMR δ [ppm].

d ^13^C chemical shifts were derived from HSQC, HMBC, and ^13^C NMR spectra.

Further experiments performed on malabarin revealed the formation of its isomeric derivatives: 17-decarboxy-malabarin^/^isomalabarin **22/22′** and 2-decarboxy-malabarin^/^isomalabarin **24/24′** as well as the less abundant 15-decarboxy-malabarin **23**, all with *m*/*z* values close to a predicted value of 669.1926 (Table S5). Similarly, the presence of 2,17-bidecarboxy-malabarin/isomalabarin (*m*/*z* close to predicted 625.2028 Da), resulting from the double decarboxylation of malabarin, was identified. HRMS experiments yielded fragmentation profiles for these derivatives analogous to those observed for globosin (Table S5). Furthermore, the same retention pattern and absorption maxima supported the identification of monodecarboxylated malabarins (λ_max_ 511, 534, and 530 nm) and 2,17-bidecarboxy-malabarin/isomalabarin (λ_max_ 514) in alignment with previous findings.^30,31,54^

Less abundant hydroxycinnamoylated gomphrenins, namely, basellin **21** and gandolin **37**, yielded analogous decarboxylated derivatives (Tables S3 and S4).

In addition, the heating of various much less abundant betacyanins recently reported in *B. alba* extracts^21^ resulted in the generation of a diverse group of derivatives. However, for the sake of simplicity, these derivatives are not included in this contribution.

In the following sections, the generation of novel decarboxylated derivatives was monitored depending on the reaction environment. This intriguing group of pigments, derived from the acylated gomphrenins, may consist of more stable compounds than gomphrenin derivatives as well as could potentially exhibit other bioactive properties.

### 3.2 Heating Studies on Gomphrenin and Its Acylated Derivatives in *B. alba* Extracts of Different Purity Levels

The influence of citric acid and EDTA on the reactivity of gomphrenin and its acylated derivatives during heating was studied for nonpurified *B. alba* extract B1 and chromatographically purified extract B2 on a strongly acidic cation-exchanger as well as extract B3 consecutively purified on cationic and ODS sorbents. In this manner, the impact of the complex *B. alba* fruit matrix on pigment degradation was observed by comparing samples of varying levels of purity. Among the acylated gomphrenins, coumaroylated gomphrenin (globosin) **13** exhibited the highest concentration in the fruits, rendering it the selected representative compound for the quantitative assessment of the acylation effect on the stability of the gomphrenin-like pigments.

This choice was based on a comparison of the impact of hydroxycinnamoyl moieties on pigment retention during heating processes, which confirmed similar stability among the four principal acylated betacyanins of *B. alba*: 6′-*O*-*E*-caffeoyl-gomphrenin (malabarin) **21**, 6′-*O*-*E*-4-coumaroyl-gomphrenin (globosin) **13**, 6′-*O*-*E*-feruloyl-gomphrenin (basellin) **29**, and 6′-*O*-*E*-sinapoyl-gomphrenin (gandolin) **37** (data not shown). Therefore, the primary experimental results are presented following the testing of gomphrenin and globosin.

**Figure 3:**
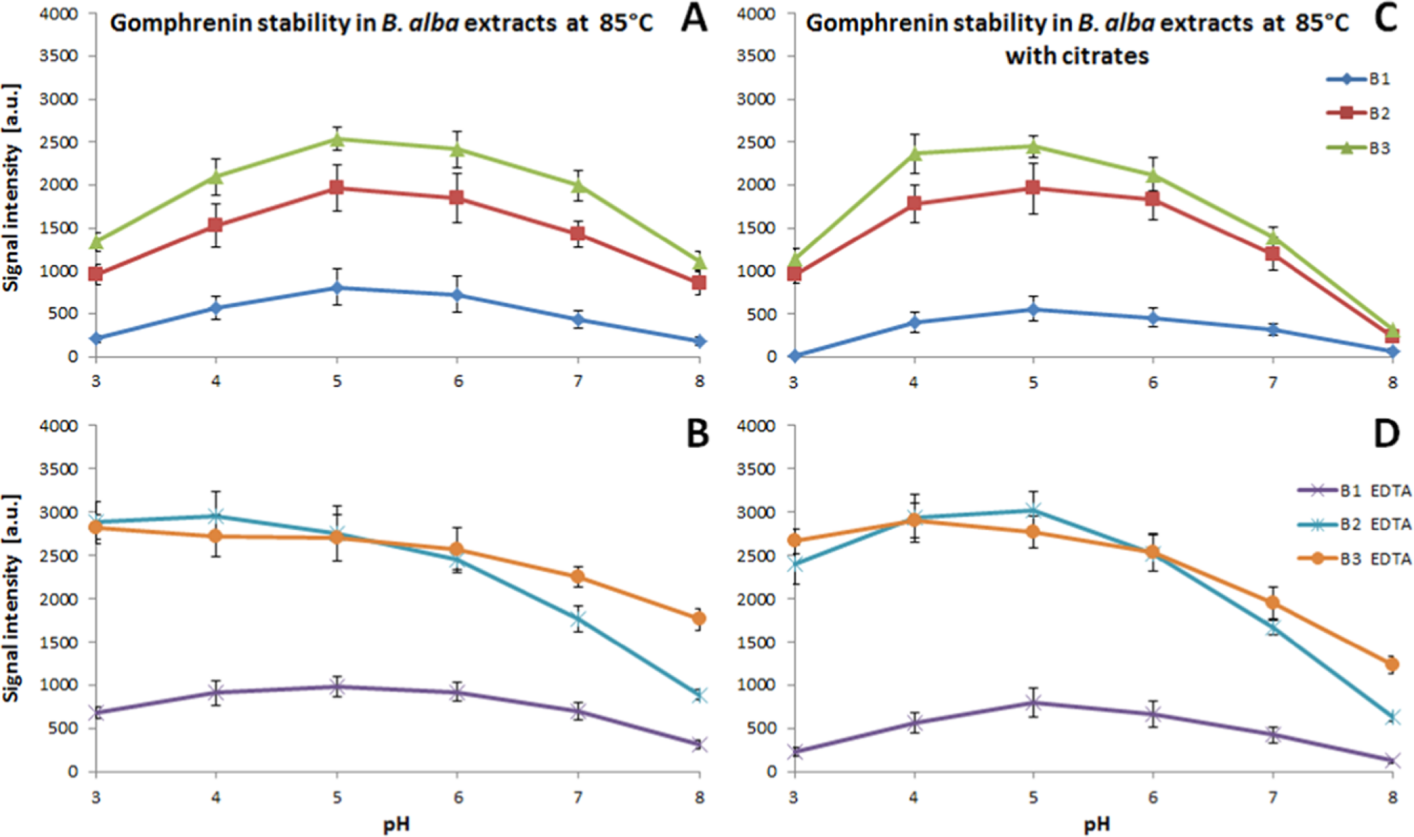
Effect of pH and the presence of citrates (A, C, D) and EDTA (B, D) on the stability of gomphrenin in reaction mixtures after 1 h of heating of 30 μM *B. alba* B1, B2, and B3 fruit extracts.

**Figure 4:**
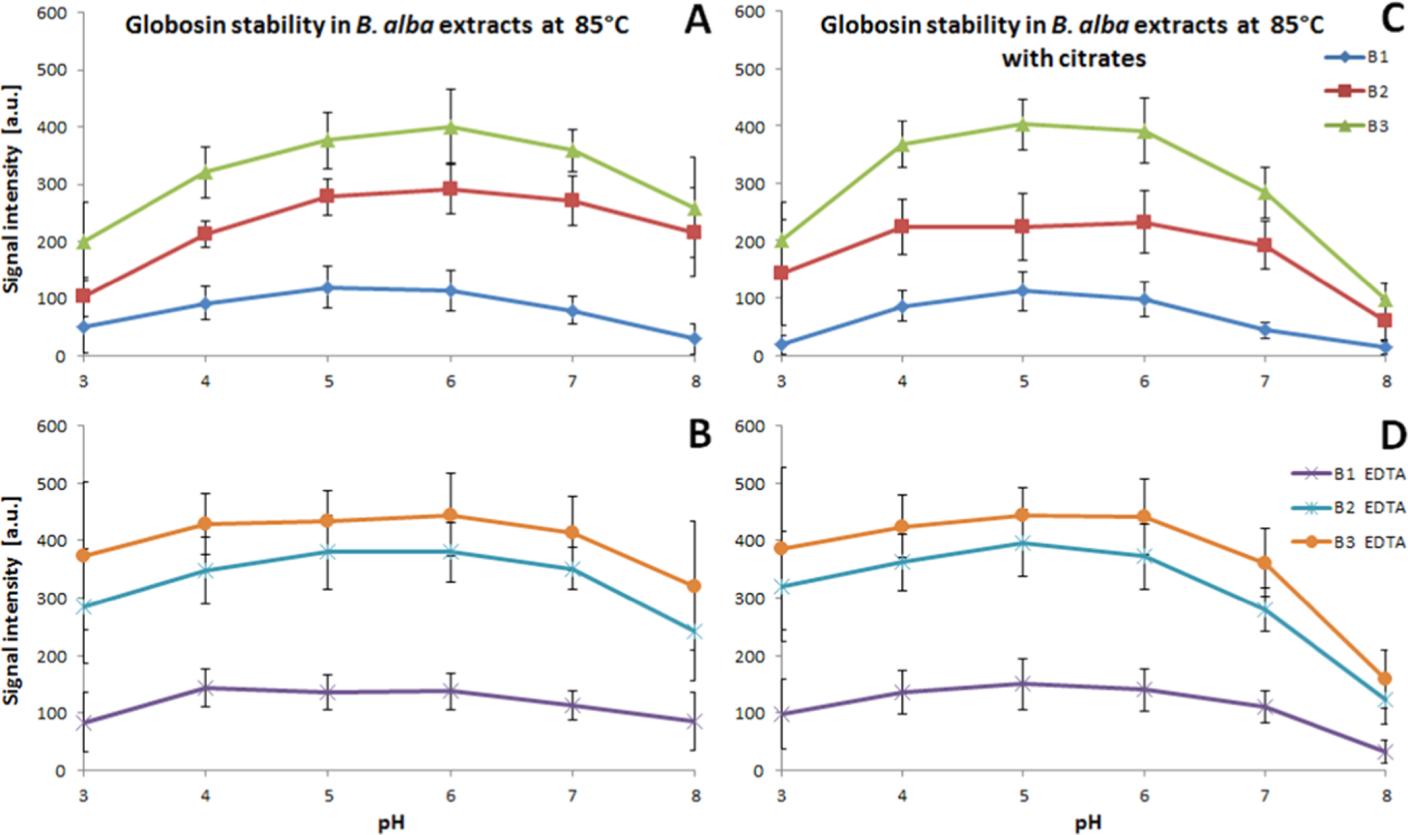
Influence of pH and the presence of citrates (A, C, D) and EDTA (B, D) on globosin stability in reaction mixtures after 1 h of heating of *B. alba* 30 μM B1, B2, and B3 fruit extracts.

The impact of heating conditions on gomphrenin **1** and globosin **13** retentions in the reaction mixtures at 85 °C is presented in Figures 3 and 4, respectively. Additional comparative results for gomphrenin obtained at different temperatures (30, 50, and 85 °C) are presented in Figures S4 and S5. Analysis of the trends indicates a higher stability of the acylated gomphrenins in all acetate/phosphate and citrate buffers as well as after the addition of EDTA (Figures 3 and 4). This phenomenon remains consistent even at various temperatures (data not shown).

The investigation into the matrix effect on pigment reactivity unveiled a significant enhancement in the stability of all betacyanins present in extract B2 following the first stage of the extract purification on a cation exchanger, regardless of the studied conditions and buffers (Figures 3, 4, S4, and S5). These findings suggest that a substantial portion of the unfavorable matrix was removed from extract B1, which likely contained a considerable quantity of reactive or catalyzing species that contributed to the pigment degradation process.

The retention of gomphrenin **1** after heating in the unpurified extract B1 at 50 and 85 °C experiences a decline to 2–3% in acetate/phosphate buffers at both pH 3 and 8 (Figures 3, S4, and S5). Under the most optimal acidity range anticipated for betacyanins (pH 5–6), the retention reaches 7–10%, which remains low in comparison to the purified extracts (ca. 20 and 30% for B2 and B3, respectively). The addition of citrates leads to a nearly complete degradation of gomphrenin at pH 3 and 8, with further reductions in retention to levels ranging from 3 to 7% at pH 5–6. However, lowering the experimental temperature to 30 °C improves the stability of gomphrenin, resulting in retention levels of 11–14% at pH 5–6 in both buffer types. Despite this improvement, significant degradation at pH 3 and 8 is still evident when citrates are present (Figures 3, S4, and S5).

The addition of EDTA to acetate/phosphate buffers enhances the retention of gomphrenin in nonpurified extract B1 at 50 and 85 °C, by ca. 10%, except pH 3 (by 5%), but at 85 °C, this increase is not as high (ca. 3%). Similar results are observed for the citrates; however, at pH 3 and 8, still a low increase is observed (by 2–3%) (Figures 3, S4, and S5).

In the case of purified extracts B2 and B3, the addition of citrates increases gomphrenin retention to a small extent in the middle pH range (5–6) but decreases it significantly at pH 8. Overall, in all of the buffers and applied temperatures, the retention differences between both the purified extract types are smaller than the differences between the nonpurified extract B1 and extract B2. Addition of EDTA decreases further the retention differences between both the purified extracts B2 and B3, and especially highly enhances the pigment retention at pH 3–4 (Figures 3, S4, and S5).

For the coumaroylated gomphrenin (globosin) **13**, most of the trends are similar to those of gomphrenin **1** except for the higher retention observed for globosin as well as higher difference between retention profiles after EDTA addition to purified extracts B2 and B3 at pH 3–6 (Figure 4). This is presumably a result of a protecting effect of the acylated moieties.

Formation of monodecarboxylated gomphrenins and monodecarboxylated globosins can be observed most conveniently by monitoring 2- and 17-decarboxy-gomphrenins/-globosins. The highest signals were detected for 17-decarboxy-gomphrenin **2** in the citrate buffers (pH 3–4) in extract B2 (Figure S6). Comparison of the compound profiles confirmed the increased generation of 17-decarboxy-gomphrenin at the highest temperature (85 °C) also for the acetate buffers as well as in extract B3. Further increase is observed for the samples with added EDTA at all applied temperatures. Almost no traces of monodecarboxylated gomphrenins were detected in the tested nonpurified extract B1. In the light of the above results obtained for gomphrenin, this is rather an effect of reactive matrix in the nonpurified *B. alba* extract, which not only does not stabilize these decarboxylation products nor the gomphrenin substrate itself but also degrades them at high extent.

For 2-decarboxy-gomphrenin **4**, significant signals were observed at elevated temperatures (50–85 °C) and especially in the citrate buffers (pH 3–4) in the extract B2 purified on cationite (Figure S6), however, only in the samples without added EDTA.

Obtained LC–MS signals for 17-decarboxy-globosin **12** and 2-decarboxy-globosin **15** were proportionally lower according to the lower content ratio of globosin and gomphrenin in the extracts (Figure S7). In contrast to the gomphrenin derivative, 17-decarboxy-globosin **12** was detected at the highest extent in both the types of purified extracts B2 and B3 at 85 °C and in both the buffer types at pH 3–4 as well as in the presence of EDTA. Furthermore, in samples of the nonpurified extract B1, especially at pH 3–5, 17-decarboxy-globosin **12** was detected, although at minute quantities, which is presumably a result of the higher stability of this acylated derivative.

The other pigment, 2-decarboxy-globosin **15**, was formed in almost all of the tested samples, including the nonpurified extracts, albeit at lower quantities than 17-decarboxy-globosin **12** (Figure S7).

### 3.3 Method for Generation of Decarboxylated Gomphrenins for Bioactivity Studies

In the preceding section, the initial findings regarding the influence of citrates and EDTA on the stability of gomphrenin-type betacyanins as well as their derivative formation in *B. alba* extracts were presented. Decarboxylated betacyanins can be obtained through partial thermal decomposition of the starting natural betacyanins; however, the efficiency of these reactions is influenced by various factors.

The nonpurified extract B1 was excluded from further tests due to its apparent degradative matrix effects on the pigments. Based on the aforementioned results, extract B2 was chosen as the pigment source because the first matrix cleanup step was found to be sufficient for the efficient generation of decarboxylated gomphrenins.

Recent reports have provided a comprehensive analysis of the generation and identification of betanin derivatives.^33,48^ In general, betanin exhibits lower activity compared to gomphrenin;^11^ thus, the intermediate products resulting from the partial decomposition of betanin, which still retain the chromophoric system, are mostly stable enough to be isolated for further investigations.

In the case of gomphrenin, only preliminary studies have been performed on its thermal^54^ and oxidative^56^ degradation, leading to initial conclusions about the potential formation of decarboxylated derivatives. Sufficient efficiency was achieved only for 17-decarboxy-derivatives.^48,54^ Given this context, our research was focused on the generation of higher quantities of 2-decarboxy-gomphrenin **4** and 2,17-bidecarboxy-gomphrenin **5** in extract B2 based on various factors among which the addition of citrates emerged as the most decisive.

**Figure 5:**
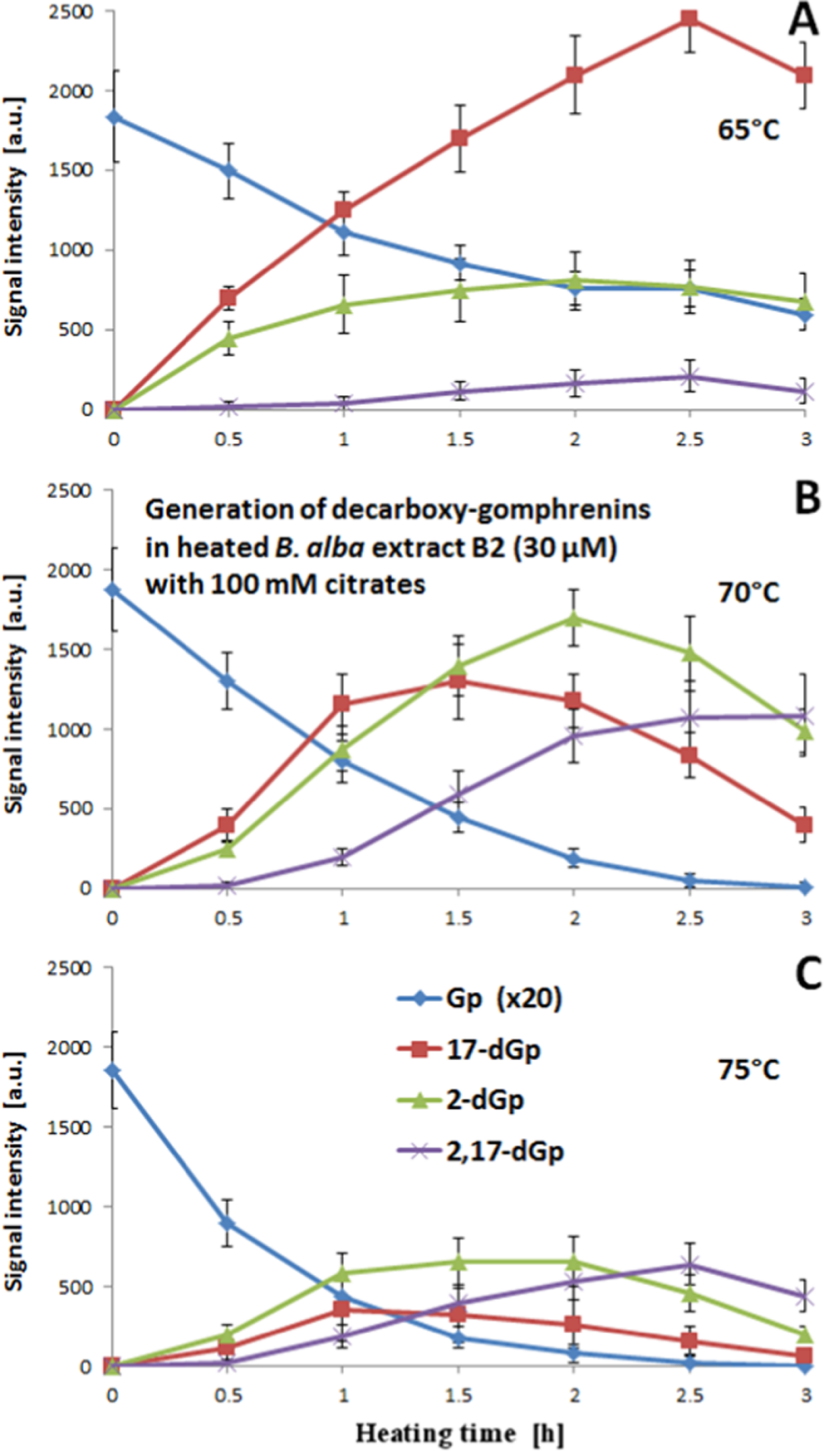
Influence of the heating temperature and time of the purified water extract B2 from *B. alba* fruits with a total concentration of betacyanins (in gomphrenin equivalents) of 30 μM in the presence of concentrated sodium citrate (100 mM) on the chemical transformation of gomphrenin and the formation of its decarboxylated derivatives at 65 (A), 70 (B), and 75 (C) °C.

The analysis of the effect of citrate addition into reaction mixtures revealed that an increasing citrate concentration favors the extensive generation of 2-decarboxy-gomphrenin **4**, and this effect is counteracted by the addition of EDTA. However, when elevated citric acid amounts (100 mM) were present, increased pigment reactivity was observed at 85 °C (data not shown), resulting in an excessively rapid degradation rate for gomphrenin **1** and its derivatives. This outcome is impractical for the preparative obtaining of decarboxylated gomphrenins. Consequently, to determine the optimal conditions for pigment formation, an additional study was conducted to select the appropriate pigment concentration and temperature for the processing of the extract B2 (Figures 5 and S8). This effort led to the narrowing of the temperature range to 65–75 °C, a significantly lower value compared to the typical 85 °C often utilized in recent experiments on betanin and gomphrenin degradation.^48,54^

Figures 5 and S6 depict the influence of applying different gomphrenin concentrations (15, 30, and 60 μM) and temperatures (65, 70, and 75 °C) on the generation of its decarboxylated derivatives **2**, **4**, and **5** in extract B2. For a convenient comparison and assessment of generation efficiency, the signals obtained for lower concentrations (15 and 30 μM) were multiplied by the factors of 4 and 2, respectively, in Figures 5 and S8A,B.

At 65 °C, the best conditions are observed for the formation of 17-decarboxy-gomphrenin, which reaches its highest concentration after 2.5 h of heating. From the presented trends (Figures 5 and S8), it is evident that the generation of 2-decarboxy-gomphrenin **4** is inefficient and too slow. In addition, the rate of 2,17-bidecarboxy-gomphrenin **5** generation is even lower because this pigment is formed from the monodecarboxy-derivatives and the gomphrenin substrate is not fully reacted.

Increasing the temperature by 5 degrees results in a significant shift in the signal ratio between 2- and 17-decarboxy-gomphrenin, which becomes higher than 1:1 after 1–2 h of heating, depending on the starting concentration of gomphrenin substrate. At higher concentrations, the 17-decarboxy-pathway is significantly hindered (Figures 5 and S8). Interestingly, the most efficient generation of 2-decarboxy-gomphrenin **4** is observed for the intermediate gomphrenin concentration (30 μM). Moreover, under these conditions, the signal from gomphrenin **1** diminishes after 3 h of heating, whereas for 2,17-bidecarboxy-gomphrenin **5**, it reaches the highest level. The highest signal for 2-decarboxy-gomphrenin **4** is observed after 2 h.

The lower rate of the decarboxylated gomphrenins’ formation at the highest tested concentration presumably results from the stabilizing effect of the abundance of pigments present in the reaction mixture in extract B2 on the gomphrenin substrate, which is not fully reacted (Figures 5 and S8). Therefore, the most optimal conditions for the generation of 2-decarboxy-gomphrenin **4** and 2,17-bidecarboxy-gomphrenin **5** in extract B2 are achieved with a medium gomphrenin concentration (30 μM) at 70 °C, with a citrate level of 100 mM and without the addition of EDTA. These conditions were applied in the process of obtaining these derivatives (Section 2.5).

### 3.4 NMR Structural Elucidation of Isolated Decarboxylated Gomphrenins

Three decarboxylated gomphrenins semisynthesized in this study were analyzed by complete 1D and 2D NMR analysis for the first time. For the aim of obtaining stable narrow signals of the zwitterionic chromophoric systems as well as appropriate solubility for the long-term two-dimensional NMR experiments, the analyses were performed in CD_3_OD acidified with TFA-*d*, except for 2-decarboxy-gomphrenin, which appeared not stable at these conditions and required D_2_O for the preparation of the analytical sample.^57^

The obtained ^1^H, ^13^C (Figures S9–S14), COSY, TOCSY, and NOESY spectra enabled us to assign basic betanidin-derived spin systems (Table 1). Detection of H-11 and H-12 protons by their distinguishable downfield signals appearing as doublets for 17- and 2,17-dGp (obtainable in acidified CD_3_OD) and broad doublet for 2-dGp (Table 2) as well as H-4 and H-7 singlets and H-15/H-14ab system indicated the presence of the typical vinyl, aromatic, and dihydropyridinic moieties characteristic for betanidin.^28,57^ A broad ^1^H NMR signal for H-18 was observed for 2-dGp (Figure S11) for freshly prepared D_2_O solutions of the pigments before the fast deuterium exchange.^28^ In the case of 17- and 2,17-dGp (Figures S9 and S13), the acidic CD_3_OD solutions enabled the observation of stable narrow signals.^21^

Broad H-2ab/H-3ab triplets in the individual ^1^H-spin system indicated the decarboxylation at carbon C-2 in 2-decarboxy-gomphrenin and 2,17-decarboxy-gomphrenin. The upfield shifts of the ^13^C signals for the C-2 and C-3 carbons additionally supported the decarboxylation at C-2 (Figures S12 and S14).

The lack of 2-decarboxylation in 17-decarboxy-gomphrenin was confirmed by the presence of the typically downfield shifted H-2 doublet of doublets at 5.21 ppm as well as strongly separated H-3ab doublet of doublets, which were overlapped by other signals in the ^1^H spectrum (Figure S9).

The 17-decarboxylation in 17-dGp and 2,17-dGp was indicated by the formation of a new H-17 proton broad doublet at δ ∼ 7.65 and 7.54 ppm, respectively, leading to the formation of the individual ^1^H-spin system of H-17 and H-18 doublets (Figures S9 and S13).

**Figure 6:**
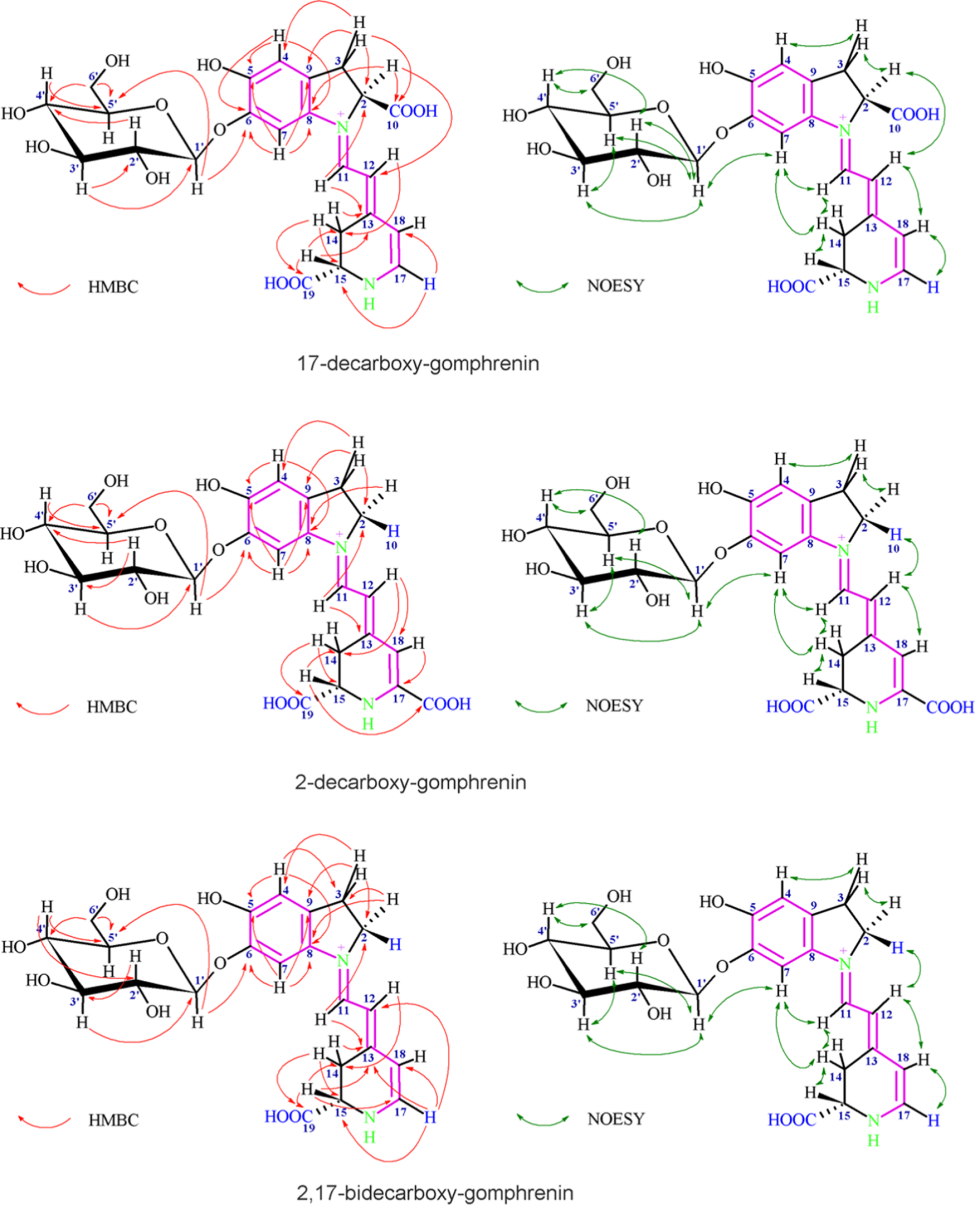
Important HMBC and NOESY NMR correlations indicating the structures of the chromophoric systems and the positions of the glycosidic in the decarboxy-gomphrenins isolated from *B. alba* L. heated extracts.

The NOESY correlations between protons H-7, H-14, and H-1′ as well as HMBC correlation between phenolic carbon C-6 and the anomeric proton H-1′ readily confirmed the substitution of the hydroxyl group at C-6 of betanidin in all of the analyzed pigments (Figure 6). In addition, the increased chemical shift difference of H-4 and H-7 (0.35 ppm) in 2-decarboxy-gomphrenin, which was measured in D_2_O, supported this substitution pattern. The shift difference for 17-dGp and 2,17-dGp was even higher, but this was presumably a consequence of NMR spectral acquisition in acidified CD_3_OD.^21^

The dihydroindolic system was unambiguously assigned by HSQC correlations of H-2 (or H-2ab), H-3ab, H-4, and H-7 with their respective carbons. Similarly assigned was the dihydropyridinic system by HSQC correlations of H-14ab, H-15, H-18, and/or not H-17.

Further correlations were obtained by HMBC technique for the dihydroindolic system in all of the three pigments (Figure 6): H-2 (or H-2ab) to C-3,8; H-3ab to C-2,4,9; H-4 to C-5,6,8; and H-7 to C-5,6,8,9 as well as for the dihydropyridinic system: H-11 to C-13; H-12 to C-14; H-14 to C-13,15; H-15 to C-13,14; and (for 17-dGp and 2,17-dGp) H-17 to C-15,18.

The principal *E*-configuration for C(12) = C(13) and the *s-trans* conformation of the betanidin dienyl system (N-1, C-11,12,13) was confirmed in the most abundant stereoisomer by NOESYcross-peaks (Figure 6) between H-7, H-11, and H-14 as well as H-2, H-12, and H-18 protons.^28,57^

The spin system of the glucopyranosyl moiety (H-1′–H-6′) was identified by TOCSY and COSY correlations. A three-bond vicinal coupling constant ^3^J_H-1′,H-2′_ 6.9–7.7 Hz indicated the β-linkage between the aglycone and glucopyranosyl moiety.

### 3.5 Antioxidant Activity of Gomphrenin and Its Derivatives

**Figure 7:**
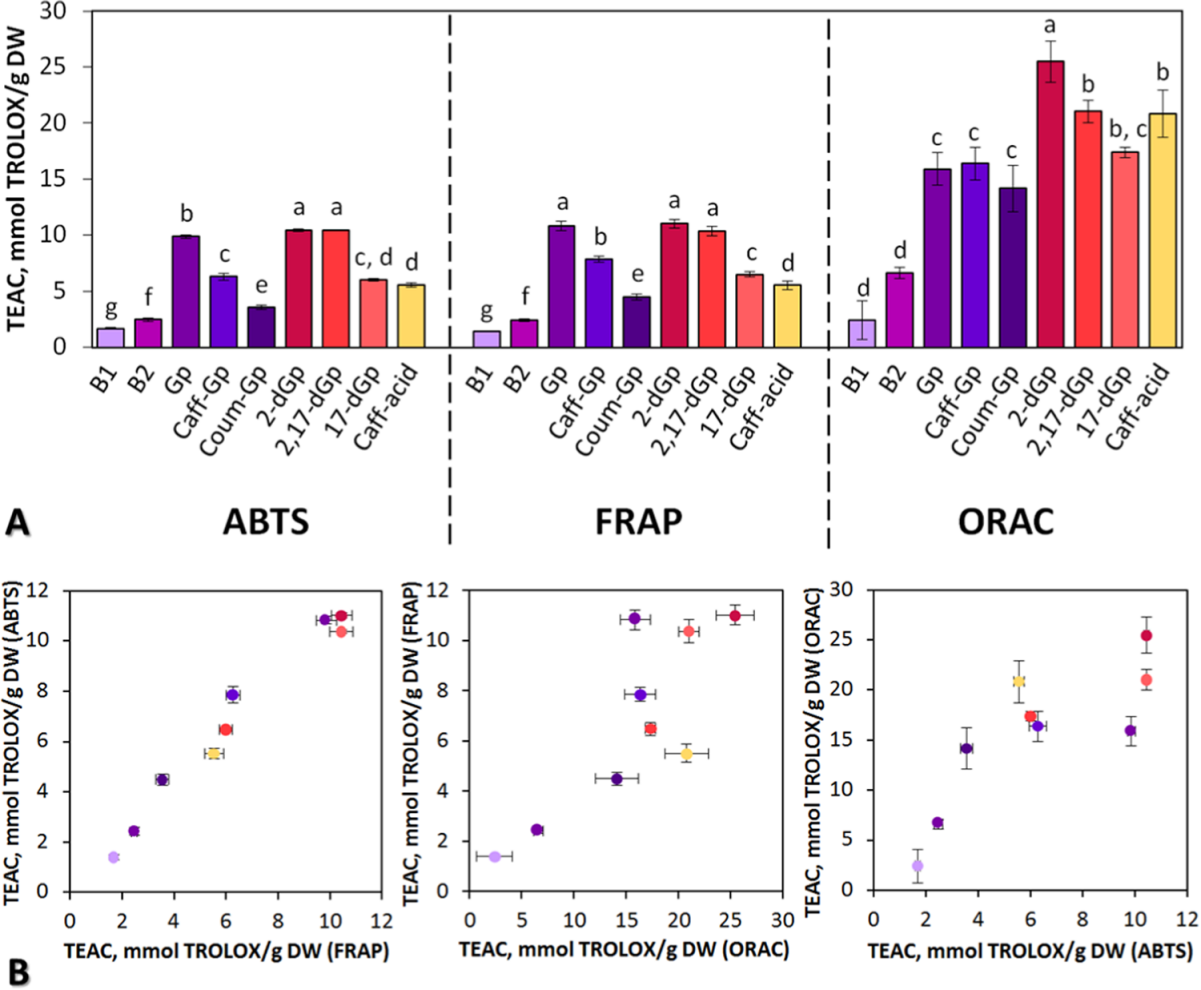
TEAC values (A) determined for *B. alba* extracts, gomphrenin, 6′-*O*-*E*-caffeoyl-gomphrenin (malabarin), 6′-*O*-*E*-4-coumaroyl-gomphrenin (globosin), 2-decarboxy-gomphrenin, 17-decarboxy-gomphrenin, and 2,17-bidecarboxy-gomphrenin and standard caffeic acid under ABTS, FRAP, and ORAC assays. Correlations between the TEAC values (B) obtained in ABTS and FRAP, FRAP and ORAC, as well as ORAC and ABTS are presented. Raw data may be consulted in Supporting Information, Table S6.

There is a growing interest in the health benefits of betalains, leading many scientists to focus on researching plants that are rich in these pigments. However, few studies have evaluated the antioxidant capacity of isolated betalains and their derivatives. *In vitro* spectrophotometric tests can be used to determine the ability of these compounds to scavenge radicals. Each test has its peculiarities; therefore, in order to measure the activities, it is necessary to carry out different tests and compare the results between them. In this study, antioxidant activity was assessed for two samples of *B. alba* plant extracts (B1 and B2) as well as five thoroughly purified pigments: 6*′*-*O*-*E*-caffeoyl-gomphrenin (malabarin, Caff-Gp) and 6′-*O*-*E*-4-coumaroyl-gomphrenin (globosin, Coum-Gp), 2-decarboxy-gomphrenin (2-dGp) 17-decarboxy-gomphrenin (17-dGp) and 2,17-bidecarboxy-gomphrenin (2,17-dGp). Malabarin, globosin, and gomphrenin were isolated from the *B. alba* extract, and decarboxylated gomphrenins were obtained by the controlled thermal decarboxylation of gomphrenin in the purified *B. alba* extract B3 at 65–75 °C. The antioxidant activity was determined using ABTS, FRAP, and ORAC methods and compared to the activity of caffeic acid as a reference substance (Figure 7 and Table S6).

When comparing the relations between the different methods, a very strong linear correlation can be observed between the results obtained for the ABTS/FRAP tests (Pearson’s correlation coefficient equals 0.99), whereas the correlation slightly deviates for the FRAP/ORAC and ORAC/ABTS comparisons. These differences in individual tests are due to the different mechanisms by which they occur.

The FRAP method is based on single electron transfer (SET) in an acidic environment (pH 3.6). On the other hand, in the ORAC method, hydrogen atom transfer (HAT) occurs. The ORAC test is performed at 37 °C and pH 7, so it corresponds to the physiological conditions in the human body from among the methods selected in this contribution. The ABTS test presents an interesting case as it can be classified as both SET- and HAT-based assays.^58^ However, from the comparison of results in the graphs for the individual tests, it can be concluded that the SET mechanism is the preferred one since the results for the ABTS test are distributed almost identically to those of the FRAP test.

Conducted ABTS and FRAP tests demonstrated significant differences (*p* < 0.05) between the TEAC values for extract B2, containing a higher betacyanin content, and the unpurified extract B1. This observation underscores the pivotal role of betacyanins in the radical scavenging activity of the tested extracts. Furthermore, the antioxidant potential of the samples increases with the level of purification. Additionally, the antioxidant properties of crude extracts are significantly higher compared to other betalain-rich plant extracts.

The results obtained for the B1 extract (1.68 ± 0.10 mmol/g DW) are higher than those obtained for β vulgaris L. extract (approximately 0.020 mmol/g DW) as reported previously^59^ in the ABTS test. The elevated results for B1 extract could likely be attributed to the distinct glycosylation position of the main betalain pigments found in the extracts. The 6-*O*-glycosylated analogues (gomphrenin and its derivatives) exhibit superior antioxidant potential compared to 5-*O*-glycosylated betacyanins.^11^

Moreover, the B1 extract also demonstrates more robust activity than the extracts from plants of *Amaranthaceae* family tested in previous studies. The results obtained for *Atriplex hortensis* var. rubra^60^ and *Amaranthus tricolor*(61) were in the range of 0.18–0.24 mmol/g DW and 0.015–0.060 mmol/g DW based on the ABTS test, respectively, and varied depending on the specific plant part.

On the contrary, higher values were obtained for extracts derived from the pulp (1.46–3.16 mmol/g DW) and peel (7.99–12.29 mmol/g DW) of prickly pear (*Opuntia ficus indica*) fruits.^62^

In the FRAP and ABTS measurements, gomphrenin (Gp) demonstrates robust antioxidant properties, surpassing those of reference compound, Caff-acid (TEAC 10.8 ± 0.4 vs 5.51 ± 0.36 mmol TE/g DW for the FRAP test and 9.87 ± 0.15 vs 5.55 ± 0.21 mmol TE/g DW for the ABTS test).

Upon comparing the results in the graphs for the individual tests in this study, it can be concluded that the SET mechanism is favored. This inference is the preferred one since the results for the ABTS test are distributed almost identically to the FRAP test.

Through resonance, the secondary amino group derived from betalamic acid conjugates with the hydroxyl group involved in the tautomeric equilibrium of the keto–enol formyl group.^63^ Electron withdrawal from the phenolic oxygen of betacyanins occurs relatively easily, and the resulting betacyanin radicals are stabilized by delocalization of the unpaired electron through the aromatic ring. This characteristic contributes to their excellent antioxidant properties.^43^ In contrast, the tested acylated gomphrenin derivatives (Caff-Gp and Coum-Gp) exhibited lower TEAC values. One hypothetical explanation for this could be that the acylation of gomphrenin contributes to higher compound stability, thereby making electron transfer into the system more challenging.^12^ However, this mechanism has not been described and requires further study. Interestingly, in both the ABTS and FRAP tests, malabarin exhibited improved antioxidant activity compared to globosin. This could be attributed to the higher number of hydroxyl groups present in the caffeic acid molecule compared to that of *p*-coumaric acid.

Particularly interesting results were also obtained for the decarboxylated derivatives, and their outcomes strongly depended on the type of assay used. In the ABTS and FRAP assays, there were no significant differences (*p* < 0.05) between the results for 2-decarboxy-gomphrenin and 2,17-decarboxy-gomphrenin, and their values did not differ from the result for gomphrenin (FRAP test) or was even higher (ABTS). In contrast, the antioxidant activity of 17-decarboxy-gomphrenin was significantly lower than that reported for both gomphrenin and the other products.

A completely different relationship can be observed for the ORAC test based on the HAT mechanism. By far, the best properties are observed for 2-decarboxy-gomphrenin (2-dGp) with a value of 25.5 ± 1.8 mmol TE/g, as well as for the other decarboxylated gomphrenins. Following previous reports, the carboxyl group is the first to lose hydrogen, followed by the hydroxyl group, and finally, the N-16 group.^64^ The proton in the dihydroindolic ring is the most acidic. The quaternary nitrogen of the indoline moiety has little influence on the antioxidant activity of this compound.^65^ The study also reports a descending order of deprotonation of betacyanins (based on betanin and betanidin) as C-17, C-15, and C-2. These properties were determined from theoretical models.^66^ The highest TEAC value for 2-dGp suggests a significant influence of 2-decarboxylation on the conjugated system in gomphrenin, which will need to be confirmed experimentally in further studies.

A characteristic change in the ORAC values obtained, compared to the other assays, is the significant increase in the antioxidant values for the acylated compounds, which are 16.4 ± 1.5 mmol TE/g for Caff-Gp and 14.1 ± 2.0 mmol TE/g for Coum-Gp. These results are comparable to the value for Gp, which is 15.9 ± 1.5 mmol TE/g. The relatively low result acquired for gomphrenin may also be due to its degradation under elevated temperature conditions during the experiment. Both decarboxylated derivatives and acylated derivatives are likely to have higher stability under experimental conditions. At the same time, the ORAC test uses peroxyl radicals, which, compared to ABTS radicals, are not sterically hindered, which may affect the results obtained. In addition, the presence of an acyl group (caffeic or 4-coumaroyl) in the pigment structure introduces an additional –OH moiety, which can act as a H donor to increase the antioxidant potential.^11^ Moreover, the results obtained for acylated gomphrenin were higher than those for acylated amaranthin (celosianin)^60^—betanin-based pigment—according to three independent tests, which is in line with previous reports.^11,67^

When comparing the relations between the different methods (Figure 5B), a very strong linear correlation can be observed between the results obtained for the ABTS/FRAP tests (Pearson’s correlation coefficient equals 0.99), whereas the correlation slightly deviates for the FRAP/ORAC and ORAC/ABTS comparisons. These differences in individual tests are due to the different mechanisms by which they occur, as well as the conditions under which the experiments are conducted.

The tested betacyanins show antioxidant activity in both SET- and HAT-based assays. However, there are varying correlations observed in each assay type, indicating that the nature of betacyanin activity depends on the mechanism involved. Furthermore, there are observed differences between gomphrenin as well as acylated and decarboxylated gomphrenins; therefore, further research on the antioxidant properties of betacyanins derived from *B. alba* fruits is suggested to gain a better understanding of their oxidation mechanism.

### 3.6 Effect of Pretreatment with Gomphrenin and Its Derivatives on the Viability of LPS-Stimulated Macrophages

**Figure 8:**
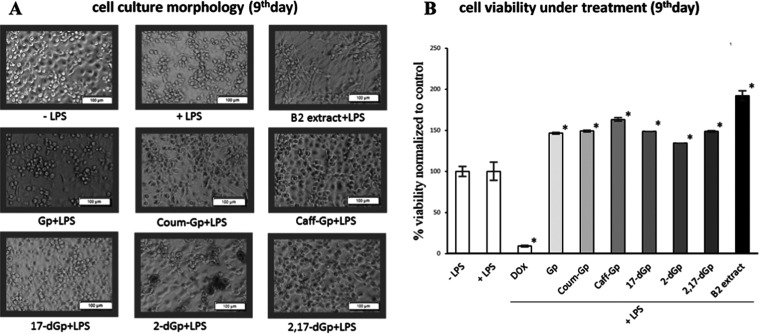
Representative micrographs (A) showing the morphology of cultured human macrophages pretreated with gomphrenins for 24 h before stimulation with LPS (0.1 μg/mL), and the effect of gomphrenins on the viability of cultured human macrophages as determined by MTT assay (B). Results are means ± SD (*n* = 3, *p < 0.05 vs. control cells, + LPS).

Several studies reported that betalains have no adverse effect on the viability of murine RAW264.7 macrophages^51^ and betaxanthins maintain a cellular redox balance and even have antiapoptotic properties in human monocyte THP-1 cell line culture.^68^ Human macrophages were treated with the selected gomphrenin pigments at a nontoxic concentration (20 μM) for 24 h prior to stimulation with 0.1 μg/mL of LPS. Negative control cells remained untreated with gomphrenins and LPS, while positive control cells were only treated with LPS. After 24 h of LPS addition, an MTT assay was performed to evaluate cell viability (Figure 8B).

Cell morphology was also visualized using an inverted microscope (Olympus IX-70 equipped with a camera, Olympus, Hamburg, Germany) (Figure 8A). The results indicate that all tested compounds support cell proliferation. The most pronounced effect was observed for extract B2, resulting in cell growth of up to 200% compared to untreated cells. The addition of LPS to untreated cells did not lead to any loss of viability. Furthermore, the morphology of human macrophages remained unchanged, with a characteristic roundish shape upon treatment with gomphrenins and LPS.

As MTT assay refers to the activity of mitochondrial pathways controlling energy production in the cell, we may conclude that tested gomphrenins rather improved cell survival and exerted a protective effect on LPS-activated macrophages. Therefore, we exclude in this study the possibility that the anti-inflammatory potential of the tested compounds in the next section was attributed to the lower viability of macrophages.

### 3.7 Effect of Gomphrenin and Its Derivatives on the Secretion of Proinflammatory Cytokines from LPS-Stimulated Human Macrophages

Macrophages, the main cells of the innate immune system, play a crucial role in regulating inflammation, tissue repair, and regeneration in the body. These cells utilize various mechanisms, including the secretion of cytokines and chemokines, to finely coordinate both innate and adaptive immune responses.^39^ The screening experiments performed in this study investigated the anti-inflammatory activity of compounds 1–6 and purified *B. alba* extract B2 by determining their effects on the release of cytokines and chemokines from human monocyte-derived macrophages. The cells were pretreated with tested samples for 24 h and then stimulated with bacterial endotoxin, LPS, for another 24 h. Previous studies have reported the significant anti-inflammatory potential of gomphrenin-rich extracts isolated from *Bougainvillea glabra*, *Gomphrena celosioides*, and *B. alba*(24,69) However, little is known about the specific effect of individual betacyanins, particularly gomphrenins, on immune cell functions. An increasing number of reports emphasize various bioactivities of extracts containing betalains,^43,70^ but the effects of acylated and decarboxylated gomphrenins remain largely unexplored.

The present study shows that the tested gomphrenin derivatives, along with the purified *B. alba* extract B2, have the capacity to modulate human macrophage function and reduce inflammation by suppressing the release of proinflammatory cytokines, including tumor necrosis factor α (TNF-α), interleukin-1β (IL-1β), and interleukin-6 (IL-6) from cells. Moreover, the tested compounds targeted distinct regulatory molecules. Specifically, Coum-Gp and Caff-Gp were found to inhibit the secretion of TNF-α, IL-1β, and IL-6 from activated macrophages. These proinflammatory cytokines induce a systemic response in the body, characterized by fever, leukocytosis, and rapid synthesis of acute phase proteins.^43,71^ Through chemotactic mechanisms, activated macrophages stimulate the migration of immune cells to the inflamed tissue, thereby exacerbating inflammation. Importantly, during acute inflammation, the body experiences oxidative stress accompanied by a significant release of TNF-α, which further stimulates the expression of MCP-1 and IL-8.^72^ Specifically, interleukin-8 (IL-8), a mediator of acute inflammatory reactions, attracts other cells of the innate immune response, such as neutrophils, to the inflamed site. The obtained data indicate that both Coum-Gp and Caff-Gp not only reduced the release of TNF-α but also effectively inhibited the chemotactic activity of macrophages by decreasing the levels of IL-8 and monocyte chemoattractant protein-1 (MCP-1/CCL2). Maintaining a balanced cytokine release from macrophages is crucial to prevent damage to infiltrated tissue cells. Therefore, the ability of Coum-Gp and Caff-Gp to restrain the secretion of multiple modulatory molecules could be important for the effective control of excessive inflammation. Additionally, it should be noted that Gp decreased the level of IL-8 in the medium but did not impact the release of TNF-α, IL-6, and IL-1β from macrophages nor the secretion of MCP-1 and CXCL1/GRO-α.

It has been demonstrated that the CXCL1 chemokine exhibits strong chemotactic properties, attracting neutrophils to inflamed tissues. Under specific conditions, an excessive influx of neutrophils into certain tissues may be related to pathological states. For example, neutrophils are considered key participants in postischemic stroke inflammation. In the ischemic brain, the prompt and abundant influx of these cells into the tissue is positively correlated with the severity of postischemic injury.^73^ Martinez et al.^74^ reported that betalains at a dosage of 100 mg/kg reduced carrageenan-induced recruitment of neutrophil migration to skin tissue in mice. We found that Coum-Gp and Caff-Gp effectively decrease the secretion of CXCL1 from activated macrophages *in vitro*. However, further studies using animal models will be needed to elucidate whether gomphrenin derivatives have the potential to regulate neutrophil recruitment to specific inflamed sites *in vivo*.

**Figure 9:**
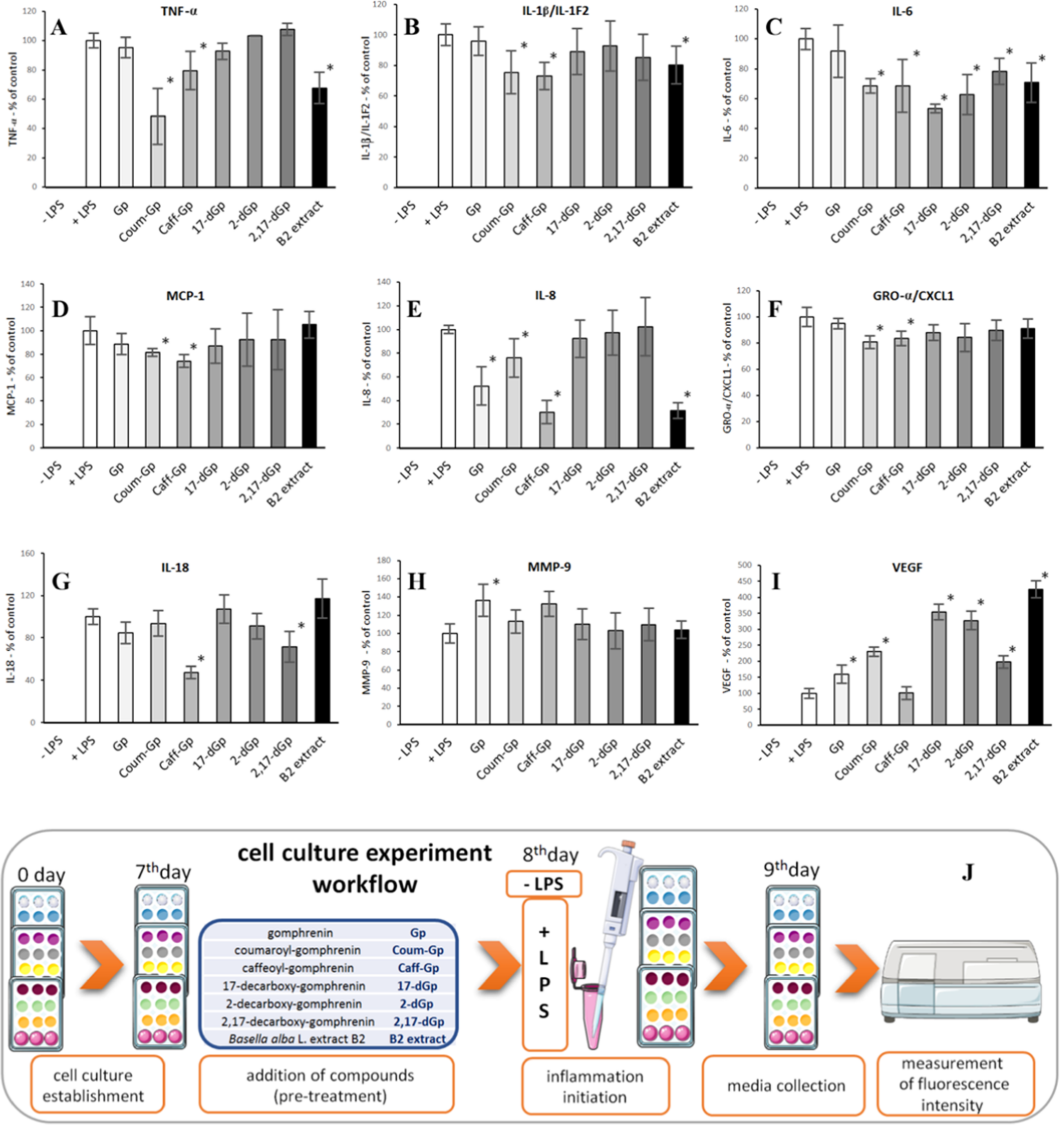
Effect of gomphrenin and its derivatives on the levels of cytokine, chemokine, and modulatory molecules released by LPS-activated macrophages determined by multiplex immunoassay using the Luminex xMAP system (A–I). Human cultured monocyte-derived macrophages were pretreated with *B. alba* extract B2 or gomphrenins at a pigment concentration of 20 μM for 24 h and then incubated with a mixture of particular compounds and LPS (0.1 μg/mL) for the next 24 h. Control cells were incubated with LPS. Cells incubated only with water, a solvent used for sample dilution, were prepared for comparison (−LPS). Bars are means ± SD (*n* = 3, *p < 0,05 vs. controls, cells + LPS). The scheme illustrating the experimental workflow is presented (J).

During inflammation, macrophages expressing proinflammatory phenotype and releasing mediators such as interleukin-18 (IL-18) can trigger various pathological processes. IL-18 acts as a costimulator, amplifying the production of interferon-γ (IFN-γ), involved in the protection of cells against infections caused by intracellular bacteria and some viruses.^75^ Although the mechanism of its action is still poorly understood, it has been discovered that the imbalanced action of IL-18 under pathological conditions *in vivo* contributes to hyperinflammation and cytokine storm, leading to, e.g., lung tissue damage and may be involved in the development of autoimmune diseases.^76^ Inhibiting of the activity of IL-18 has recently gained intense research attention and has been proposed as a novel therapeutic target for various disorders such as rheumatic diseases and infections, including Covid-19 (severe acute respiratory syndrome coronavirus 2, SARS-CoV-2).^77^ In fact, IL-18 has been identified as a diagnostic marker for acute respiratory distress syndrome, chronic obstructive pulmonary disease, and sepsis-induced multiorgan injury.^75^ Our findings indicate that both Coum-Gp and the acylated/decarboxylated gomphrenin derivative 2,17-dGp inhibited the secretion of the proinflammatory cytokine IL-18. In contrast, Gp and *B. alba* extract B2 did not show a statistically significant effect on the IL-18 secretion. As shown in Figure 9, decarboxylated gomphrenins affected experimental inflammation, but they expressed different modes of action than Coum-Gp and Caff-Gp. 17-dGp and 2-dGp specifically decreased IL-6 secretion without altering other regulatory proteins.

As mentioned above, IL-6 is a highly pyrogenic cytokine that exerts systemic reactions in the body. Therefore, selective targeting of IL-6 secretion by decarboxylated gomphrenin derivatives is of interest and may be useful in designing precise modulators of inflammation, but more advanced studies are required.

Functionally, in human body, LPS-activated macrophages have a strong phagocytotic capacity to remove debris and apoptotic cells, promoting tissue repair within wound microenvironment. Beyond their role in pathogen defense, macrophages also play a key role in various physiological processes, such as wound healing, where multiple mechanisms are triggered to replace damaged tissues with new cells.^78^ In this context, the activation of macrophages results in the increased release of paracrine and autocrine mediators of tissue repair.^40^ During wound healing, physiological processes such as extracellular matrix degradation are carried out by metalloproteinases (MMPs), including metalloproteinase-9 (MMP-9), which promotes tissue remodeling.^79^ In such conditions, vascular endothelial growth factor (VEGF) secreted by macrophages regulates tissue neovascularization by the induction of angiogenesis. Among tested compounds, Gp increased the release of MMP-9 and Gp, Coum-Gp decarboxylated and acylated gomphrenins promoted the release of VEGF from macrophages. Further experiments will elucidate the potency of the gomphrenins in tissue repair.

The obtained data show that the high biological activity of tested compounds is attributed to their modulatory effect on immune cells. The *in vitro* screening experiments revealed that gomphrenins demonstrated strong anti-inflammatory properties in the culture of LPS-activated human macrophages. In general, gomphrenin derivatives were more active than *B. alba* extract B2. The study showed that Coum-Gp and Caff-Gp targeted different regulatory molecules than decarboxylated gomphrenins.

Notably, the anti-inflammatory action of Coum-Gp and Caff-Gp was associated with the inhibition of the secretion of IL-6, IL-1β, and TNF-α, the main signaling molecules triggering systemic inflammation in the human body.

This study provided the first preliminary evidence that decarboxylated gomphrenins can selectively influence the secretion of cytokine Il-6. The tested compounds affected the release of chemokines (e.g., MCP-1) from activated macrophages, modulating the chemotactic activity of immune cells and their ability to release tissue remodeling mediators. Moreover, Gp derivatives expressed the capacity to regulate wound microenvironment signaling molecules, which, combined with the precise inhibition of IL-6 secretion (Figure 9) and lack of toxic effects, suggests the possibility of application of these compounds *in vivo*. It was well studied that the increased production of proinflammatory molecules in the body promotes the transition of the inflammatory response into the chronic state, which, in turn, contributes to the development of numerous diseases. The present results suggest that gomphrenins have therapeutic potential, and we hope that these compounds can support novel precise treatments of inflammation-associated diseases.

In conclusion, the *in vitro* screening experiments revealed that tested gomphrenin-based pigments demonstrated strong anti-inflammatory properties in the culture of LPS-activated human macrophages. In general, gomphrenin derivatives were more active than *B. alba* extract B2. The study showed that *p*-coumaroylated gomphrenin (globosin) and caffeoylated-gomphrenin (malabarin) targeted different regulatory molecules than decarboxylated gomphrenins. Notably, the anti-inflammatory action of globosin and malabarin was associated with the inhibition of the secretion of IL-6, IL-1β, and TNF-α, the main signaling molecules triggering systemic inflammation in the human body. This study provided the first evidence that decarboxylated gomphrenins can selectively influence the secretion of cytokine Il-6.

Our preliminary findings suggest that gomphrenins, including acylated and decarboxylated derivatives, obtained from *B. alba* may not only restrain inflammation by decreasing the secretion of proinflammatory cytokines from human monocyte-derived macrophages but can also promote mechanisms important for wound healing and tissue damage repair.

## Supporting Information Available

Image of *B. alba* plants grown in a greenhouse (Figure S1), chemical structures of studied pigments (Figures S2 and S3), effect of pH and the presence of the stability of gomphrenin in heated reaction mixtures (Figures S4 and S5), influence of pH, citrates, and EDTA on the formation of decarboxylated derivatives of gomphrenin and globosin in reaction mixtures after heating of purified *B. alba* B2 and B3 fruit extracts at 85 °C (Figures S6 and S7), influence of heating time of the purified *B. alba* fruit aqueous extract B2 in the presence of concentrated citric acid on the chemical transformation of gomphrenin and the formation of its decarboxylated (Figure S8), ^1^H and ^13^C NMR spectra of decarboxylated derivatives of gomphrenin (Figures S9–S14), chromatographic, spectrophotometric, and mass spectrometric data of the analyzed gomphrenin, malabarin, basellin, and gandolin pigments present in *B. alba* fruit extracts B1, B2, and B3 submitted to heating at 85 °C (Tables S1–S4), high-resolution mass spectrometric data obtained for novel decarboxylated acylated gomphrenins (Table S5), and TEAC values determined for the pigments in the ABTS, FRAP, and ORAC tests (Table S6) (PDF)
