# Supplementary material for: Basella alba L. (Malabar Spinach) as an Abundant Source of Betacyanins: Identification, Stability, and Bioactivity Studies on Natural and Processed Fruit Pigments
Source: J Agric Food Chem. 2024 Feb 1;72(6):2943–62. doi: 10.1021/acs.jafc.3c06225 (PMC10870984; doi:10.1021/acs.jafc.3c06225)
Supplement: Supplementary file 1 — jf3c06225_si_001.pdf [file jf3c06225_si_001.pdf]

Basella alba L. (Malabar Spinach) as an Abundant Source of Betacyanins: Identification, Stability, and Bio-activity Studies on Natural and Processed Fruit Pigments

Supplementary material

Katarzyna Sutor-Świeży[a,*], Renata Górska[a], Agnieszka Kumorkiewicz-Jamro[a,b,c], Ewa Dziedzic[d], Monika Bieniasz[d], Przemysław Mielczarek[e,f], Łukasz Popenda[g], Karol Pasternak[h], Małgorzata Tyszka-Czochara[i], Monika Baj-Krzyworzeka[j], Monika Stefańska[j], Przemysław Błyszczuk[j] and Sławomir Wybraniec[a,*]

[a] Department C-1, Faculty of Chemical Engineering and Technology, Cracow University of Technology, ul. Warszawska 24, 31-155, Krakow, Poland

[b] South Australian Health and Medical Research Institute, Adelaide 5000 SA, Australia

[c] Faculty of Health and Medical Sciences, University of Adelaide, Adelaide 5000 SA, Australia

[d] Faculty of Biotechnology and Horticulture, University of Agriculture in Krakow, al. 29 Listopada 54, 31-425, Krakow, Poland

[e] Department of Analytical Chemistry and Biochemistry, Faculty of Materials Science and Ceramics, AGH University of Science and Technology, al. Adama Mickiewicza 30, 30-059, Krakow, Poland

[f] Laboratory of Proteomics and Mass Spectrometry, Maj Institute of Pharmacology, Polish Academy of Sciences, ul. Smętna 12, 31-343, Krakow, Poland

[g] NanoBioMedical Centre, Adam Mickiewicz University, ul. Wszechnicy Piastowskiej 3, 61-614, Poznan, Poland

[h] Institute of Bioorganic Chemistry, Polish Academy of Sciences, ul. Noskowskiego 12/14, Poznan, 61-704, Poland

[i] Faculty of Pharmacy, Jagiellonian University Medical College, ul. Medyczna 9, Krakow, 30-688, Poland

[j] Jagiellonian University Medical College, Faculty of Medicine, Department of Clinical Immunology, Institute of Pediatrics, 30-688, Kraków, Poland

* Corresponding authors:

Katarzyna Sutor-Świeży

katarzyna.sutor@doktorant.pk.edu.pl

ORCID: 0000-0001-7354-5376

Sławomir Wybraniec

slawomir.wybraniec@pk.edu.pl

ORCID: 0000-0002-1263-4188

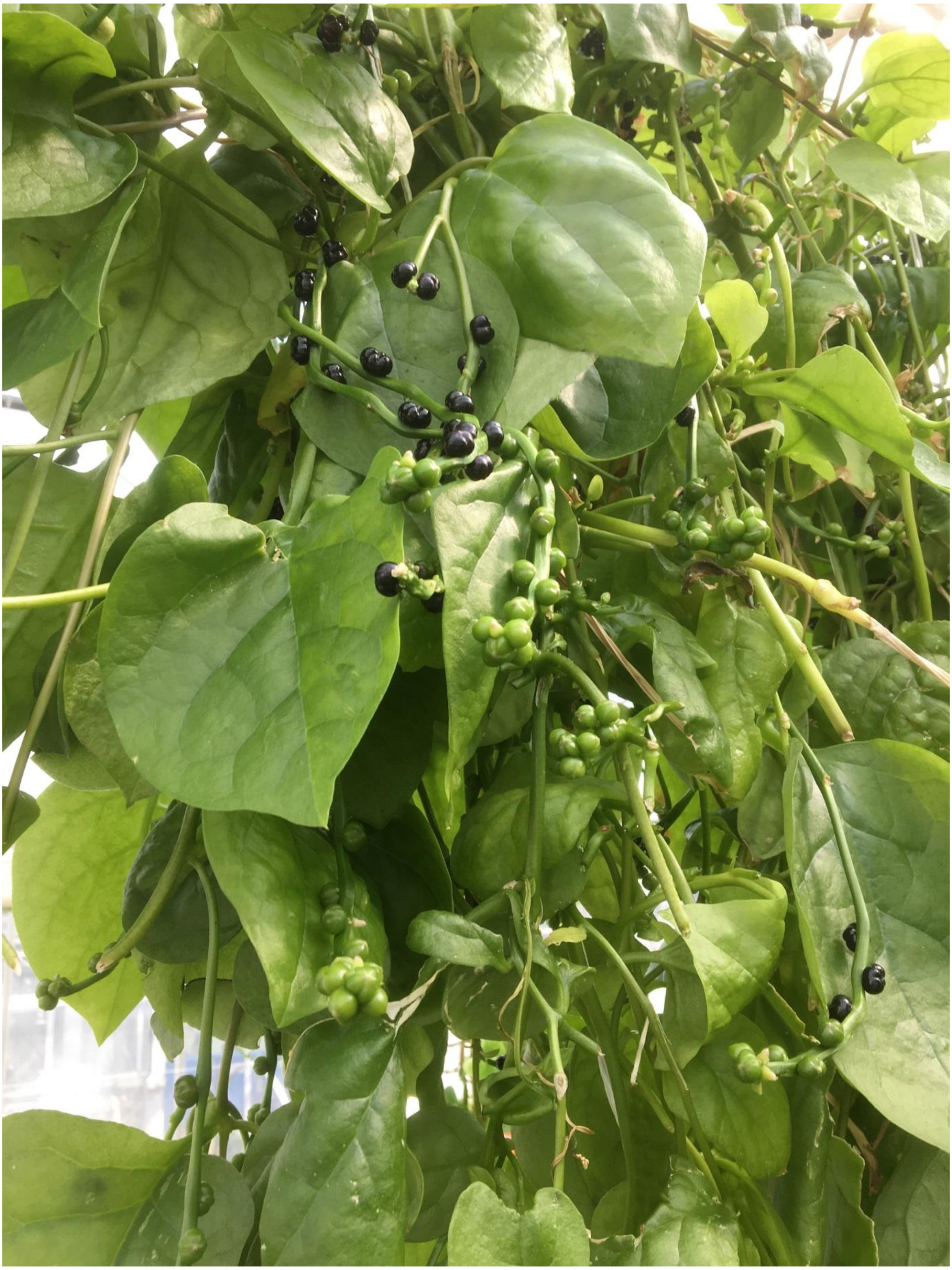

Figure S1. Image of *Basella alba* L. plant grown in a greenhouse.

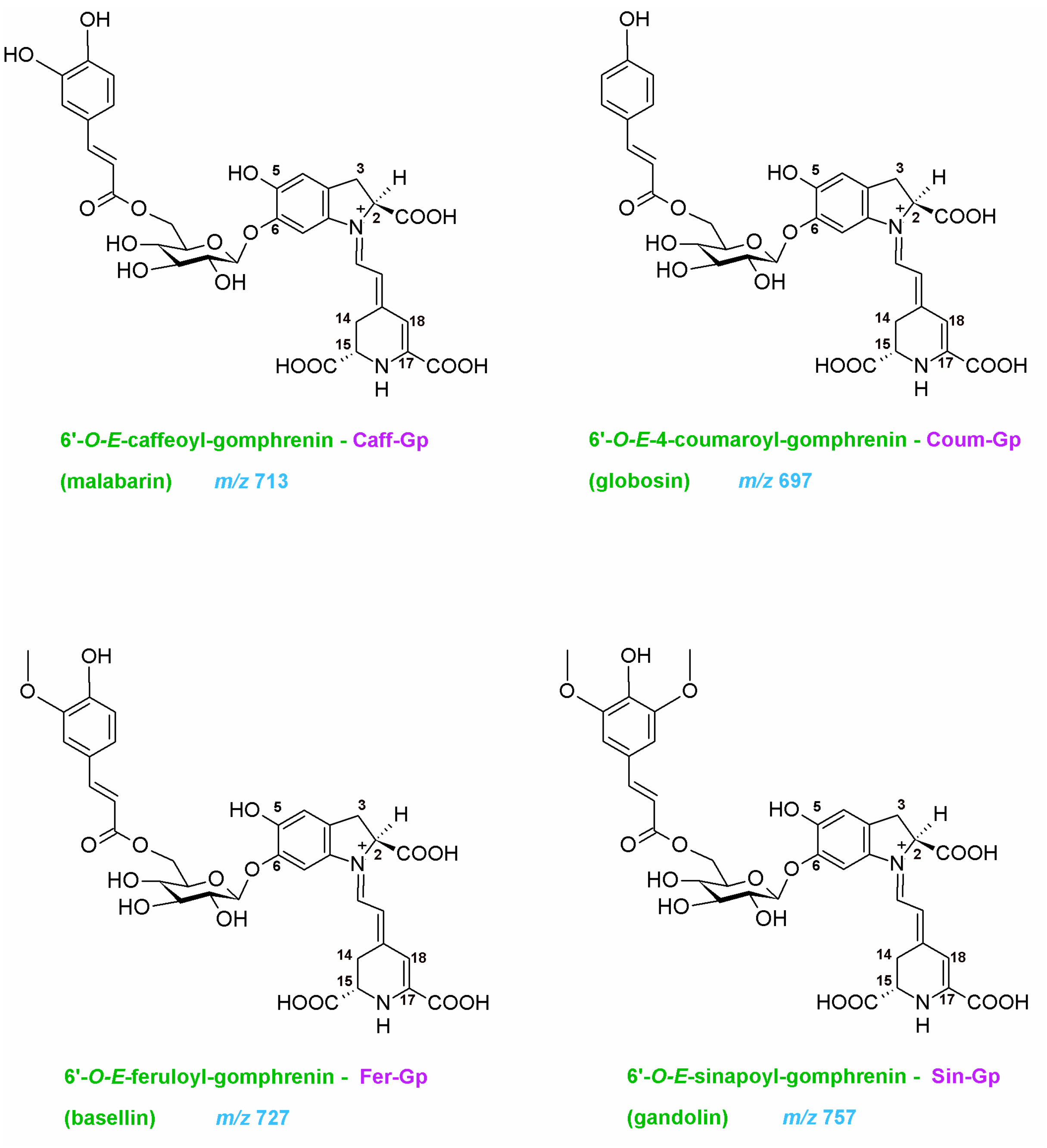

Figure S2. Chemical structures of acylated gomphrenins isolated from *B. alba* fruit extracts submitted to heating studies.

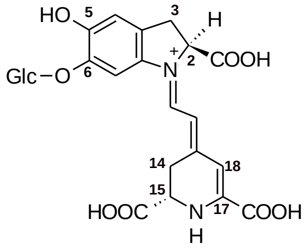

gomphrenin **1**

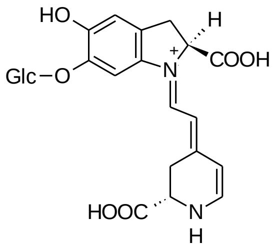

17-decarboxy-gomphrenin **2**

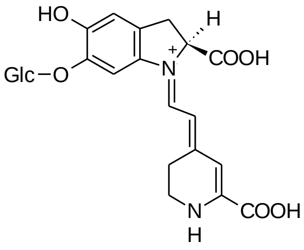

15-decarboxy-gomphrenin **3**

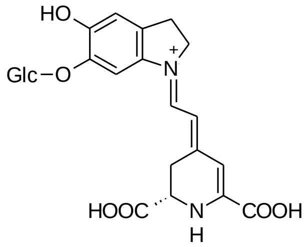

2-decarboxy-gomphrenin **4**

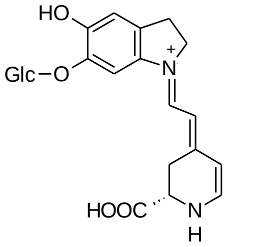

2,17-bidecarboxy-gomphrenin **5**

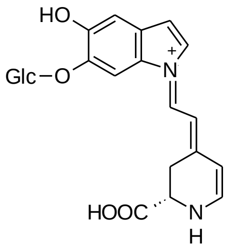

2,17-bidecarboxy-xangomphrenin **6**

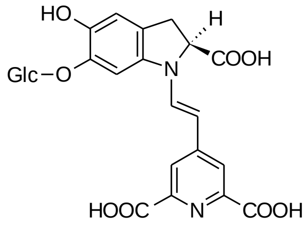

neogomphrenin **7**

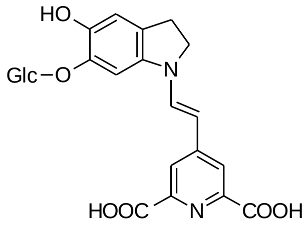

2-decarboxy-neogomphrenin **8**

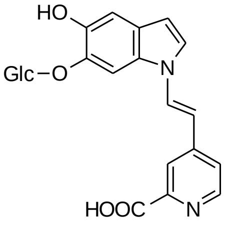

2,17-bidecarboxy-xanneogomphrenin **9**

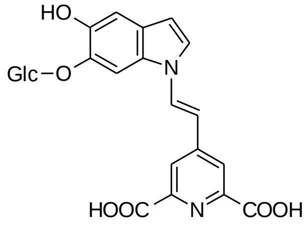

2-decarboxy-xanneogomphrenin **10**

Figure S3. Chemical structures of detected non-acylated gomphrenin derivatives in *B. alba* fruit extracts B1, B2 and B3 submitted to heating experiments.

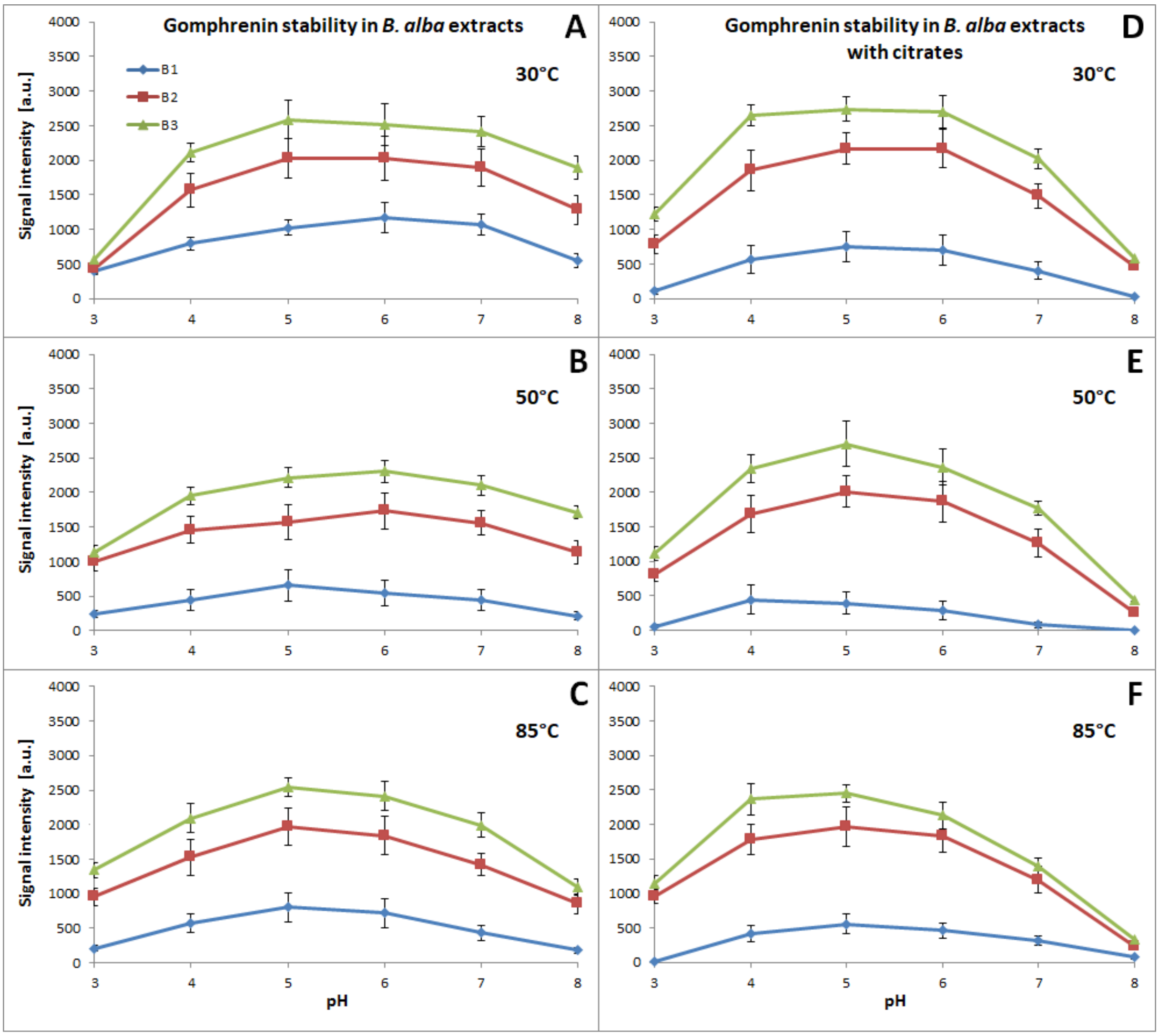

Figure S4. Effect of pH (A-F) and the presence of citrates (D, E and F) on the stability of gomphrenin in reaction mixtures after 72 h (A and D), 8 h (B and E) or 1 h (C and F) heating of 30 µM *B. alba* B1, B2, and B3 fruit extracts at 30, 50 and 85 °C, respectively.

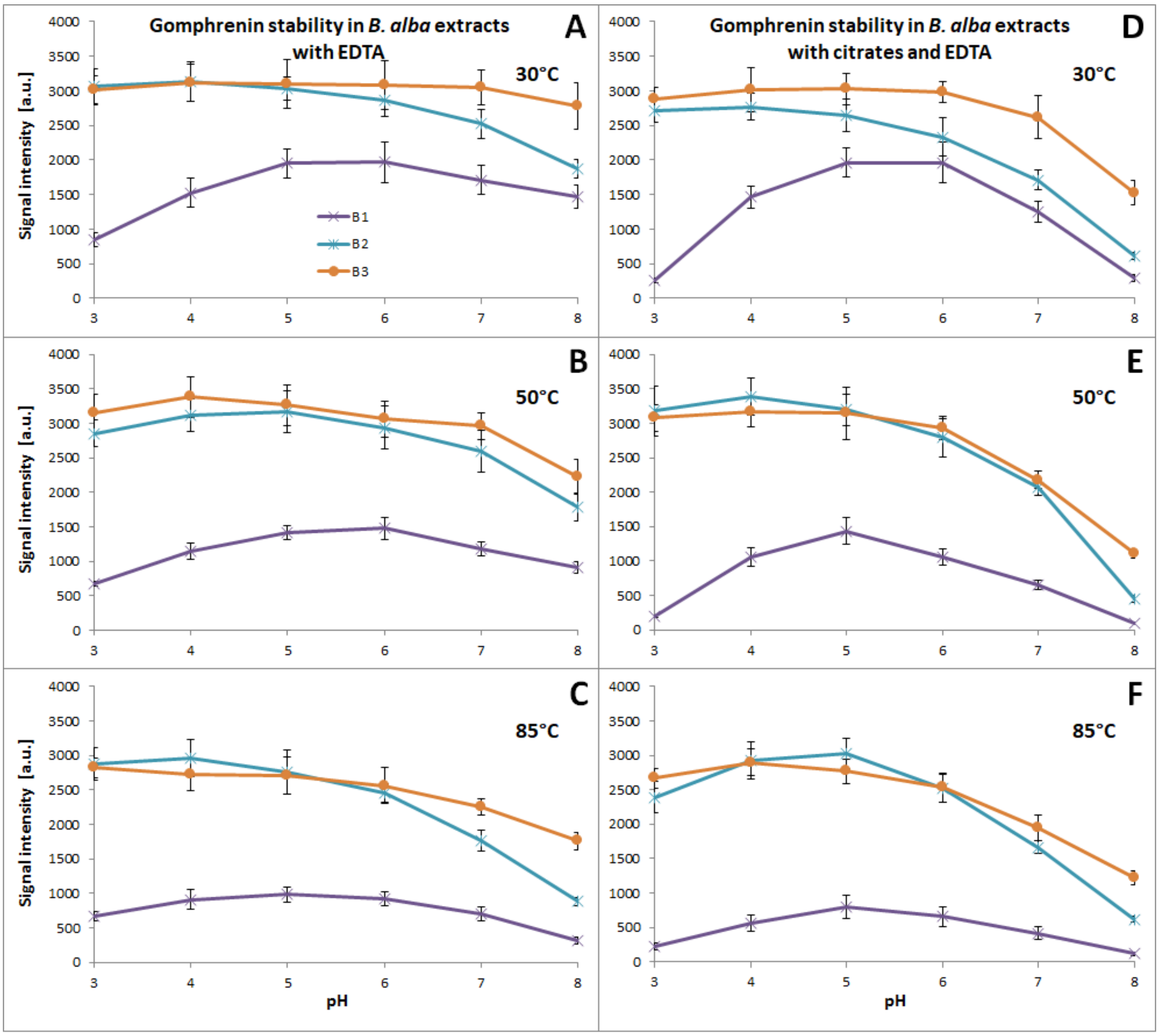

Figure S5. Effect of pH (A-F) and the presence of citrates (D, E and F) on the stability of gomphrenin in reaction mixtures containing EDTA after 72 h (A and D), 8 h (B and E) or 1 h (C and F) heating of 30 µM *B. alba* B1, B2, and B3 fruit extracts at 30, 50 and 85 °C, respectively.

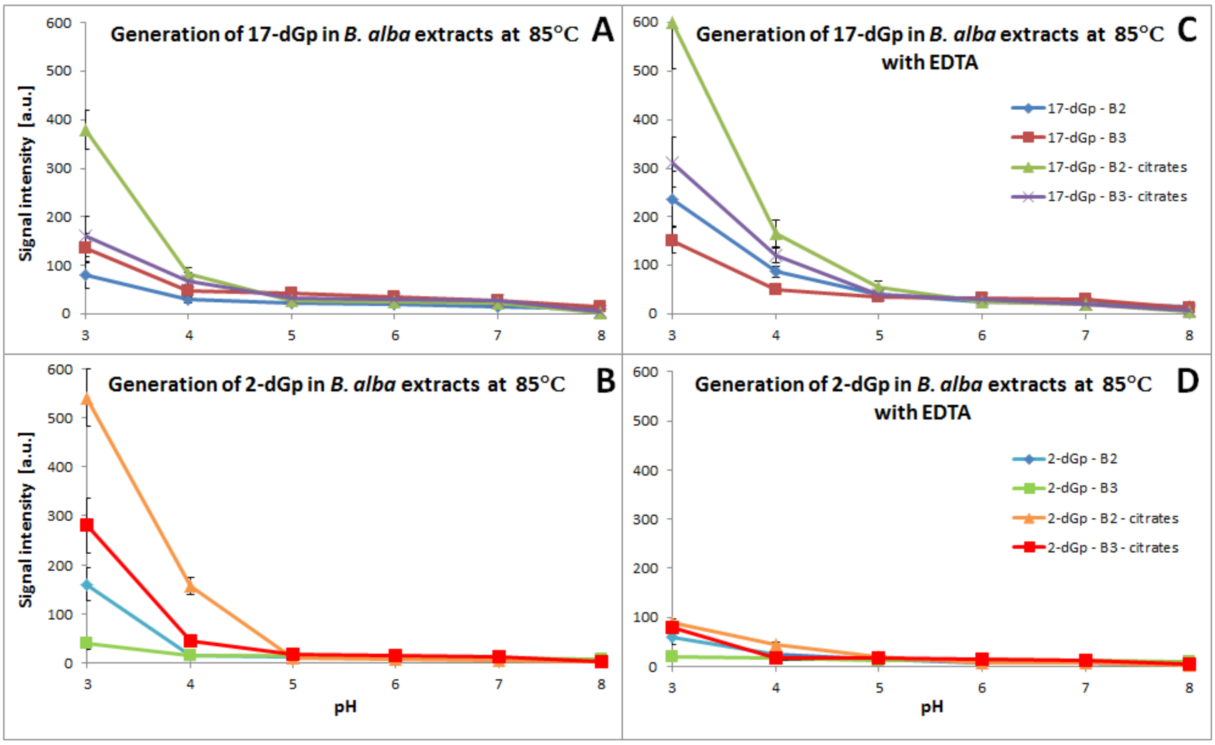

Figure S6. Influence of pH, citrates and EDTA on the formation of decarboxylated gomphrenin derivatives in reaction mixtures after heating (1 h) of purified *B. alba* B2 and B3 fruit extracts at 85 °C.

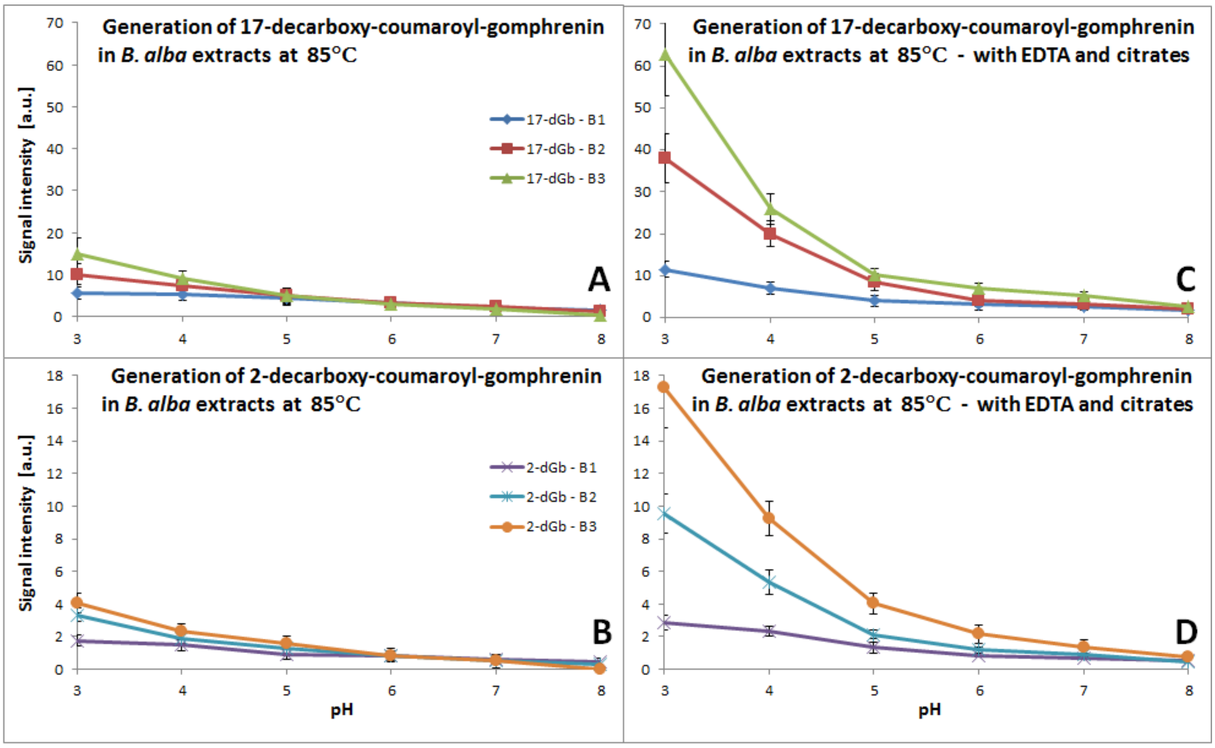

Figure S7. Influence of pH, citrates and EDTA on the formation of decarboxylated coumaroyl-gomphrenin (globosin) derivatives in reaction mixtures after heating (1 h) of purified *B. alba* B2 and B3 fruit extracts at 85 °C.

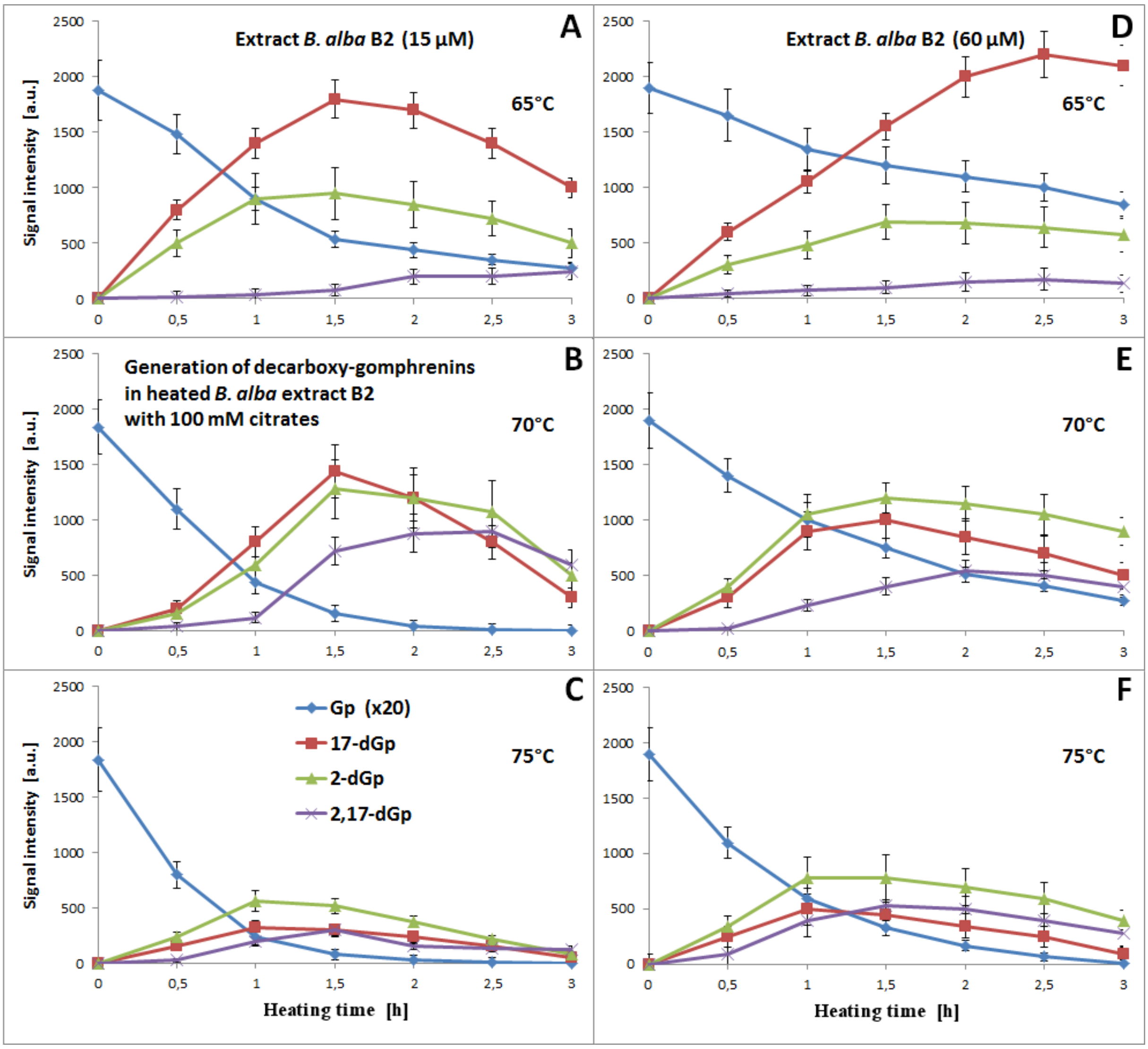

Figure S8. Influence of heating time of purified *B. alba* fruit aqueous extract B2 (betacyanin total concentration - 15 and 60 µM) in the presence of concentrated citric acid (100 mM) on the chemical transformation of gomphrenin and the formation of its decarboxylated derivatives at 65, 70 and 75 °C.

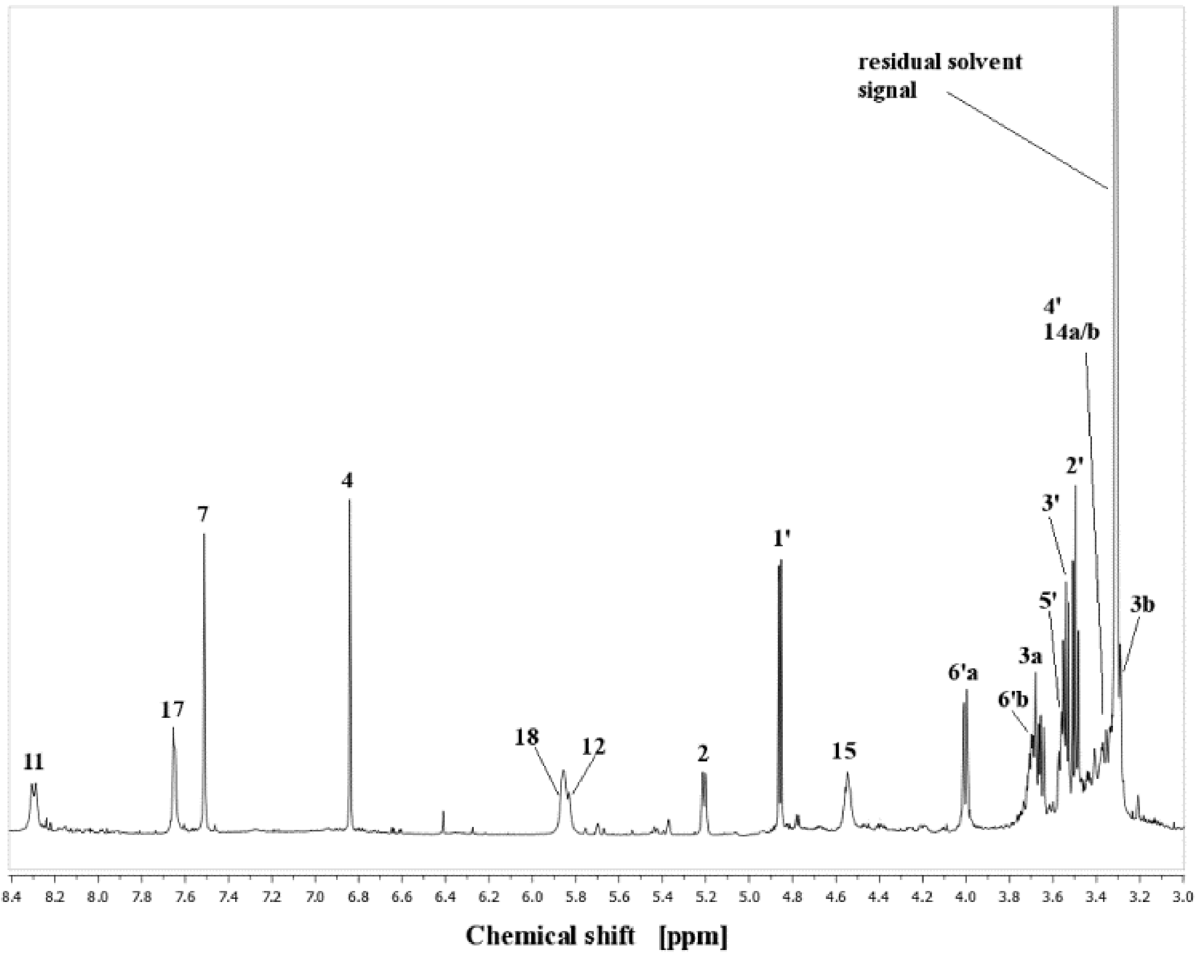

Figure S9. ^{1}H NMR spectrum of 17-decarboxy-gomphrenin (CD_3OD/d-TFA, 295 K).

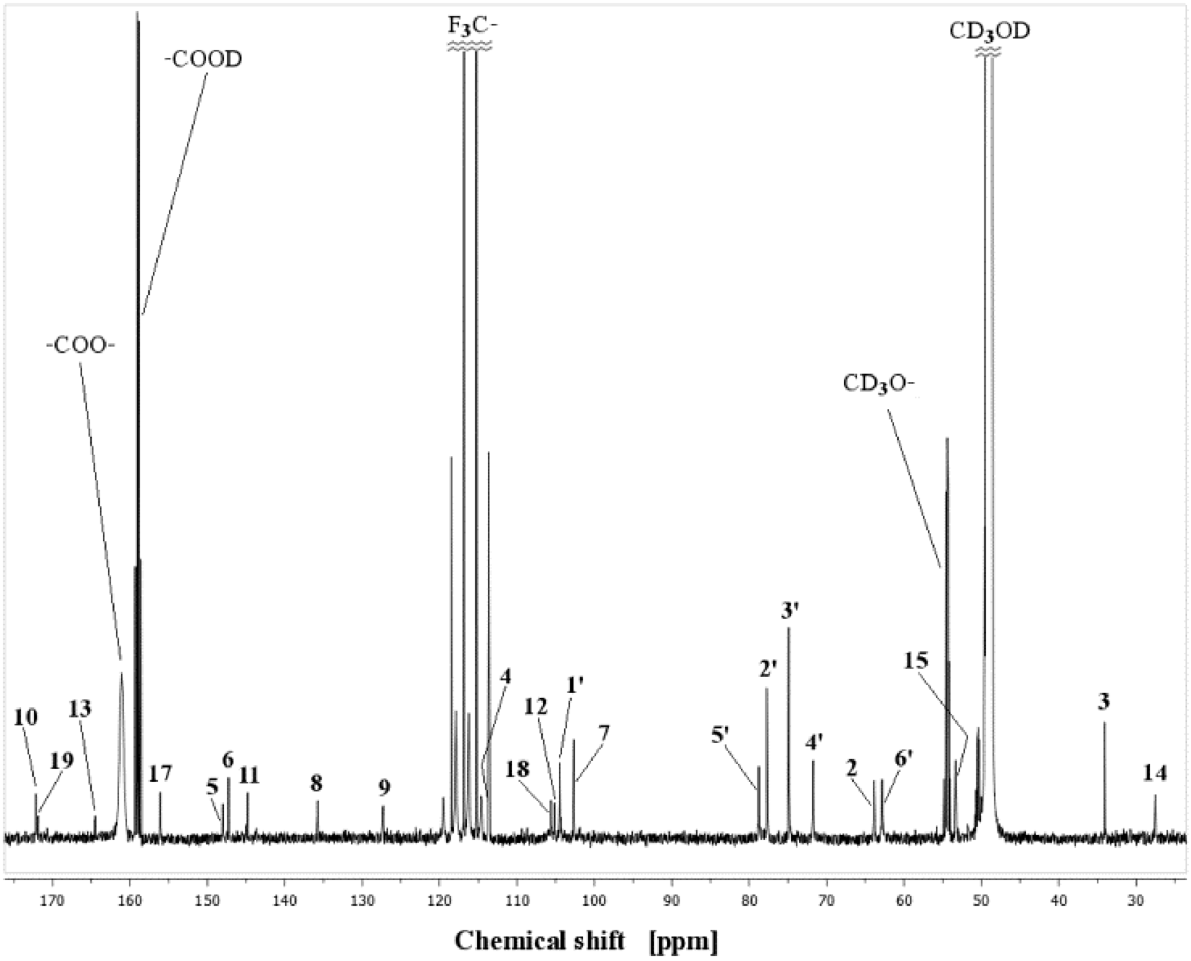

Figure S10. ^{13}C NMR spectrum of 17-decarboxy-gomphrenin (CD_3OD/d-TFA, 295 K).

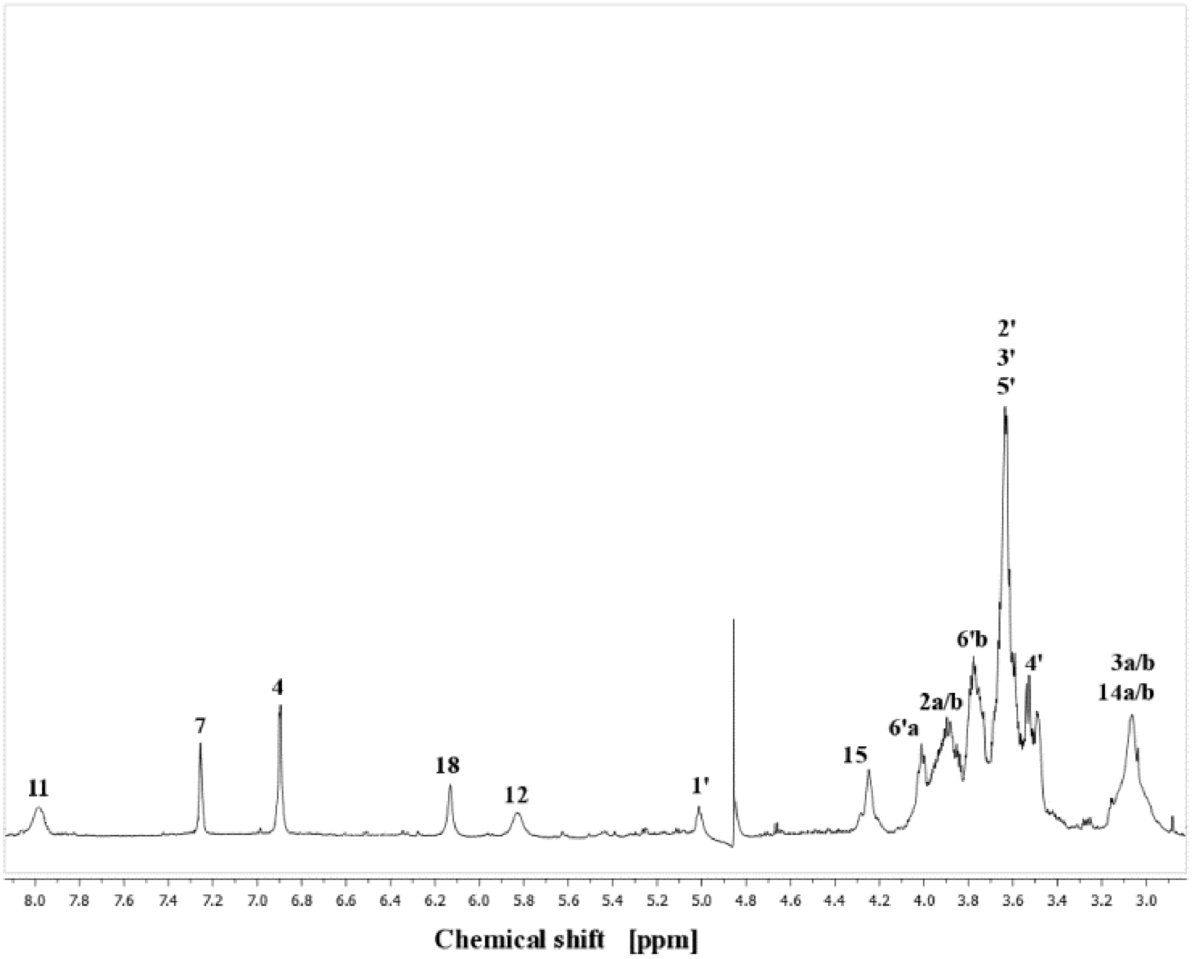

Figure S11. ^1H NMR spectrum of 2-decarboxy-gomphrenin (D_2O, 295 K).

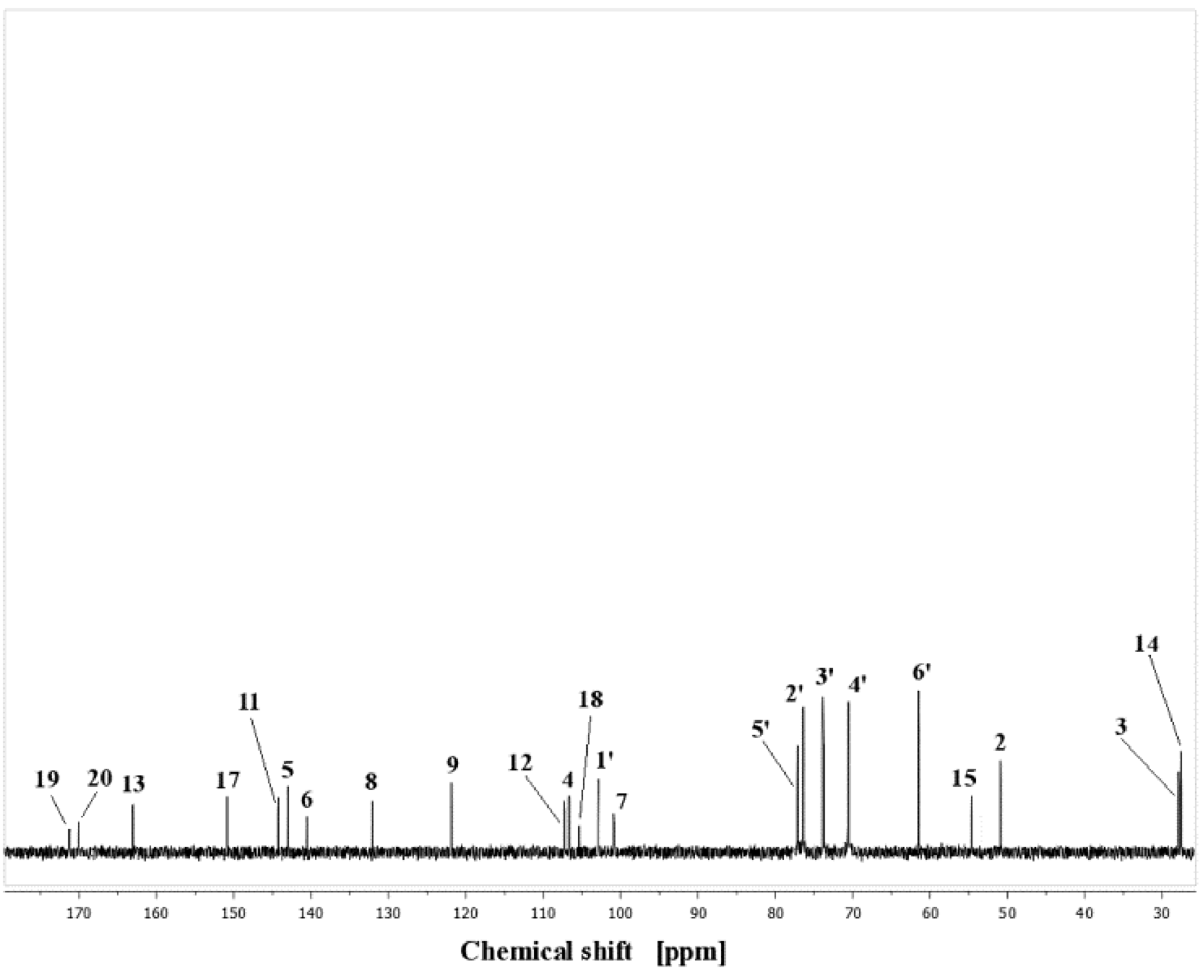

Figure S12. ^{13}C NMR spectrum of 2-idecarboxy-gomphrenin (D_2O, 295 K).

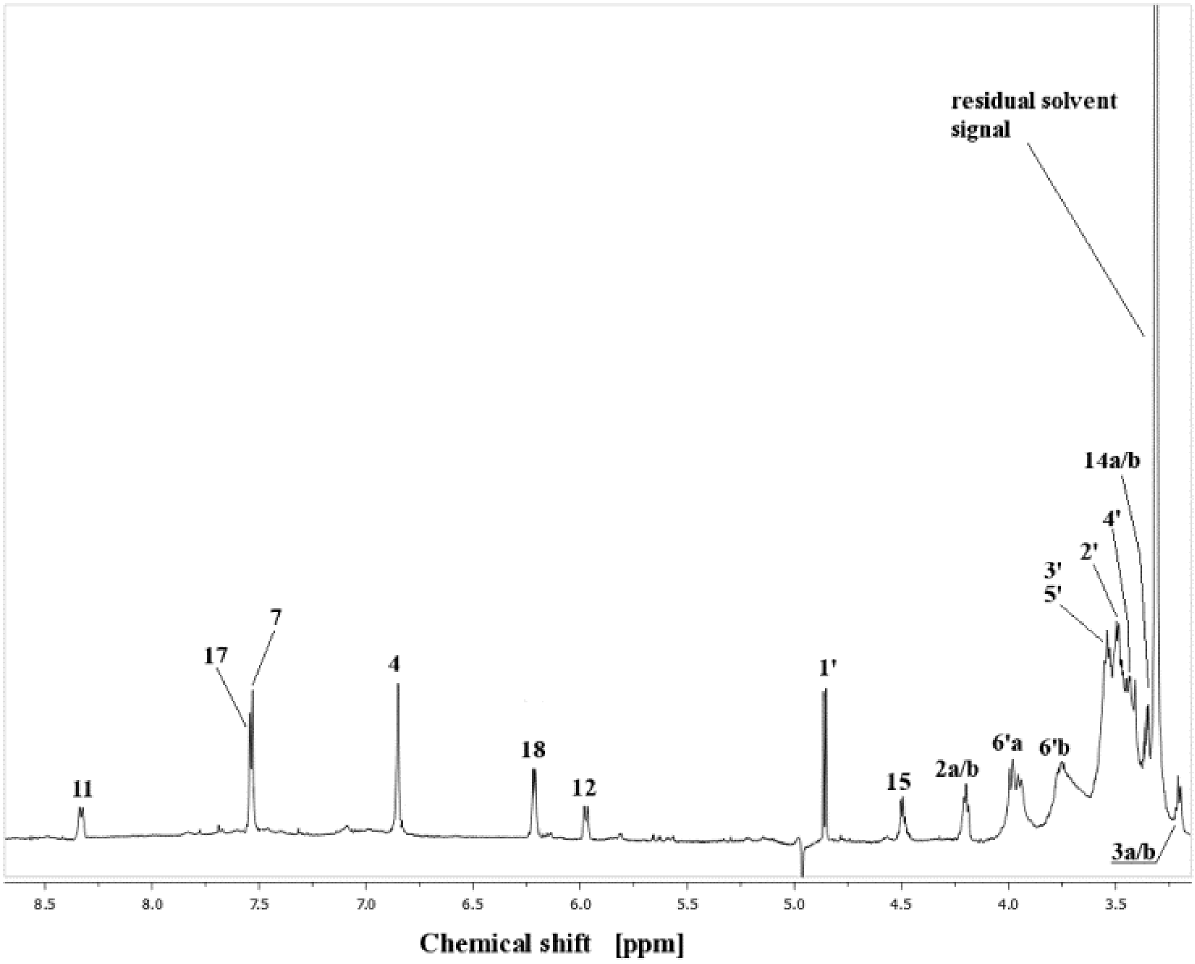

Figure S13. ^{1}H NMR spectrum of 2,17-bidecarboxy-gomphrenin (CD_3OD/d-TFA, 295 K).

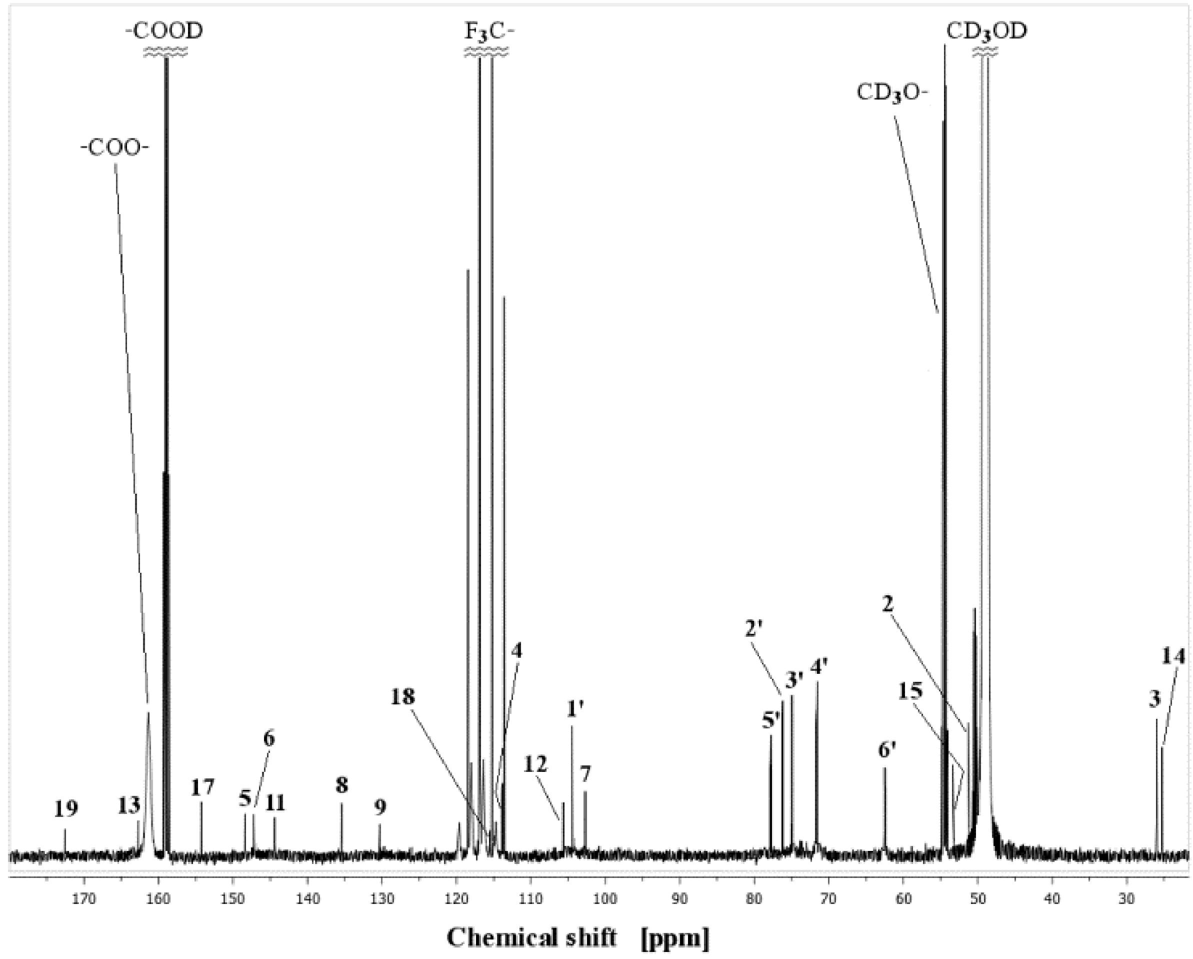

Figure S14. ^{13}C NMR spectrum of 2,17-bidecarboxy-gomphrenin (CD_3OD/d-TFA, 295 K).

Table S1. Chromatographic, Spectrophotometric and Mass Spectrometric Data of the Analyzed Gomphrenin-based Betacyanins Present in *B. alba* Fruit Extracts B1, B2 and B3 Submitted to Heating at 85 ºC.

No.	Compound	Abbreviation	R_t [min]	λ_{max} [nm]	*m/z*	*m/z* MS/MS of $[M+H]^+$
1	gomphrenin	Gp	7.9	539	551	389
1′	isogomphrenin	IGp	8.5	539	551	389
2	17-decarboxy-gomphrenin	17-dGp	8.1	509	507	345
2′	17-decarboxy-isogomphrenin	17-dIGp	8.8	509	507	345
3	15-decarboxy-gomphrenin [a]	15-dGp	9.4	530	507	345
4	2-decarboxy-gomphrenin	2-dGp	9.6	534	507	345
4′	2-decarboxy-isogomphrenin	2-dIGp	9.7	534	507	345
5	2.17-bidecarboxy-gomphrenin	2,17-dGp	10.2	510	463	301
5′	2,17-bidecarboxy-isogomphrenin	2,17-dIGp	10.3	510	463	301
6/6′	2,17-bidecarboxy-xangomphrenin [a]	2,17-dXGp	8.0	465	461	299
7	neogomphrenin [a]	NGp	11.3	483	549	387
8	2-decarboxy-neogomphrenin [a]	2-dNGp	12.4	-[b]	505	343
9	2,17-bidecarboxy-xanneogomphrenin [a]	2,17-dXNGp	9.6	412	459	297
10	2-decarboxy-xanneogomphrenin [a]	2-dXNGp	12.1	424	503	341

[a] – tentatively identified ; [b] –λ_{max} not determined due to co-elution

Table S2. Chromatographic, Spectrophotometric and Mass Spectrometric Data of the Analyzed Malabarin-based Betacyanins Present in *B. alba* Fruit Extracts B1, B2 and B3 Submitted to Heating at 85 ºC.

No.	Compound	Abbreviation	R_t [min]	λ_{max} [nm]	*m/z*	*m/z* MS/MS of $[M+H]^+$
20	*cis*-malabarin	cisMb	11.6	545	713	551; 389
20′	*cis*-isomalabarin	cisIMb	12.0	545	713	551; 389
21	malabarin	Mb / Caff-Gp	12.6	545	713	551; 389
21′	isomalabarin	IMb / Caff-IGp	13.4	545	713	551 ; 389
22	17-decarboxy-malabarin [a]	17-dMb	12.6	511	669	507; 345
22′	17-decarboxy-isomalabarin [a]	17-dIMb	13.2	511	669	507; 345
23	15-decarboxy-malabarin [a]	15-Mb	14.1	532	669	507; 345
24	2-decarboxy-malabarin [a]	2-dMb	13.1	538	669	507; 345
24′	2-decarboxy-isomalabarin [a]	2-dIMb	13.7	538	669	507; 345
25	2,17-bidecarboxy-malabarin [a]	2.17-dMb	13.5	513	625	463; 301
25′	2.17-bidecarboxy-isomalabarin [a]	2,17-dIMb	13.7	513	625	463; 301
26	2-decarboxy-xanmalabarin [a]	2-dXMb	14.4	459	667	505; 343
27	neomalabarin [a]	NMb	15.0	485	711	549; 387

[a] – tentatively identified

Table S3. Chromatographic, Spectrophotometric and Mass Spectrometric Data of the Analyzed Basellin-based Betacyanins Present in *B. alba* Fruit Extracts B1, B2 and B3 Submitted to Heating at 85 ºC.

No.	Compound	Abbreviation	R_t [min]	λ_{max} [nm]	*m/z*	*m/z* MS/MS of $[M+H]^+$
28	*cis*-basellin	cisBs	13.5	545	727	551; 389
28′	*cis*-isobasellin	cisIBs	13.8	545	727	551; 389
29	basellin	Bs / Fer-Gp	14.3	545	727	551; 389
30	17-decarboxy-basellin [a]	17-dBs	14.3	512	683	507; 345
31	2-decarboxy-basellin [a]	2-dBs	14.9	538	683	507; 345
30′	17-decarboxy-isobasellin [a]	17-dIBs	15.1	512	683	507; 345
29′	isobasellin	IBs / Fer-IGp	15.3	545	727	551; 389
32	2,17-bidecarboxy-basellin [a]	2,17-dBs	15.0	514	639	463; 301
31′	2-decarboxy-isobasellin [a]	2-dIBs	15.5	538	683	507; 345
32′	2,17-bidecarboxy-isobasellin [a]	2,17-dIBs	15.5	514	639	463; 301
33	15-decarboxy-basellin [a]	15-dBs	15.8	533	683	507; 345
34	2-decarboxy-xanbasellin [a]	2-dXBs	16.2	461	681	505; 343
35	neobasellin [a]	NBs	16.5	485	725	549; 387

[a] – tentatively identified

Table S4. Chromatographic, Spectrophotometric and Mass Spectrometric Data of the Analyzed Gandolin-based Betacyanins Present in *B. alba* Fruit Extracts B1, B2 and B3 Submitted to Heating at 85 ºC.

No.	Compound	Abbreviation	R_t [min]	λ_{max} [nm]	*m/z*	*m/z* MS/MS of $[M+H]^+$
36	*cis*-gandolin	cisGd	12.8	545	757	551; 389
36′	*cis*-isogandolin	cisIGd	13.4	545	757	551; 389
37	gandolin	Gd /Sin-Gp	14.1	545	757	551; 389
38	17-decarboxy-gandolin [a]	17-dGd	14.1	512	713	507; 345
39	2-decarboxy-gandolin [a]	2-dGd	14.4	539	713	507; 345
38′	17-decarboxy-isogandolin [a]	17-dIGd	15.0	512	713	507; 345
37′	isogandolin	IGd / Sin-IGp	15.2	545	757	551; 389
39′	2-decarboxy-isogandolin [a]	2-dIGd	15.2	539	713	507; 345
40	neogandolin [a]	NGd	16.4	485	755	549; 387

[a] – tentatively identified

Table S5. High-resolution Mass Spectrometric Data Obtained by Analysis of *B. alba* by the Orbitrap System Indicating the Presence of Novel Decarboxylated Acylated Gomphrenins as well as Their Fragmentation Ions

Compounds and fragmentation ions[a]	$[M+H]^+$ molecular formula	$[M+H]^+$ observed	$[M+H]^+$ predicted	Error [mDa]	Error [ppm]
17-decarboxy-globosin	$C_{32}H_{33}N_2O_{13}$	653.1975	653.1977	-0.2	-0.31
nl: - CO_2	$C_{31}H_{33}N_2O_{11}$	609.2078	609.2079	-0.1	-0.16
nl: - Coum	$C_{23}H_{27}N_2O_{11}$	507.1613	507.1609	0.4	0.79
nl: - Coum/Glc	$C_{17}H_{17}N_2O_6$	345.1079	345.1081	-0.2	-0.58
nl: - Coum/Glc/CO_2	$C_{16}H_{17}N_2O_4$	301.1180	301.1183	-0.3	-1.00
nl: - Coum/Glc/2CO_2	$C_{15}H_{17}N_2O_2$	257.1280	257.1285	-0.5	-1.94
nl: - Coum/Glc/2CO_2/2H	$C_{15}H_{15}N_2O_2$	255.1124	255.1128	-0.4	-1.57
2-decarboxy-globosin	$C_{32}H_{33}N_2O_{13}$	653.1972	653.1977	-0.5	-0.77
nl: - CO_2	$C_{31}H_{33}N_2O_{11}$	609.2083	609.2079	0.4	0.66
nl: - Coum	$C_{23}H_{27}N_2O_{11}$	507.1607	507.1609	-0.2	-0.39
nl: - Coum/Glc	$C_{17}H_{17}N_2O_6$	345.1082	345.1081	0.1	0.29
nl: - Coum/Glc/CO_2	$C_{16}H_{17}N_2O_4$	301.1188	301.1183	0.5	1.66
nl: - Coum/Glc/2CO_2	$C_{15}H_{17}N_2O_2$	257.1290	257.1285	0.5	1.94
15-decarboxy-globosin	$C_{32}H_{33}N_2O_{13}$	653.1983	653.1977	0.6	0.92
nl: - Coum	$C_{23}H_{27}N_2O_{11}$	507.1615	507.1609	0.6	1.18
nl: - Coum/Glc	$C_{17}H_{17}N_2O_6$	345.1084	345.1081	0.3	0.87
2,17-bidecarboxy-globosin	$C_{31}H_{33}N_2O_{11}$	609.2077	609.2079	-0.2	-0.33
nl: - Coum	$C_{22}H_{27}N_2O_9$	463.1708	463.1711	-0.3	-0.65
nl: - Coum/Glc	$C_{16}H_{17}N_2O_4$	301.1181	301.1183	-0.2	-0.66
nl: - Coum/Glc/CO_2	$C_{15}H_{17}N_2O_2$	257.1282	257.1285	-0.3	-1.17
nl: - Coum/Glc/CO_2/2H	$C_{15}H_{15}N_2O_2$	255.1125	255.1128	-0.3	-1.18
17-decarboxy-malabarin	$C_{32}H_{33}N_2O_{14}$	669.1928	669.1926	0.2	0.30
nl: - CO_2	$C_{31}H_{33}N_2O_{12}$	625.2029	625.2028	0.1	0.16
nl: - Caff	$C_{23}H_{27}N_2O_{11}$	507.1611	507.1609	0.2	0.39
nl: - Caff/Glc	$C_{17}H_{17}N_2O_6$	345.1085	345.1081	0.4	1.16
nl: - Caff/Glc/CO_2	$C_{16}H_{17}N_2O_4$	301.1180	301.1183	-0.3	-1.00
nl: - Caff /Glc/2CO_2/2H	$C_{15}H_{15}N_2O_2$	255.1126	255.1128	-0.2	-0.78
2-decarboxy-malabarin	$C_{32}H_{33}N_2O_{14}$	669.1925	669.1926	-0.1	-0.15
nl: - CO_2	$C_{31}H_{33}N_2O_{12}$	625.2030	625.2028	0.2	0.32
nl: - Caff	$C_{23}H_{27}N_2O_{11}$	507.1606	507.1609	-0.3	-0.59
nl: - Caff/Glc	$C_{17}H_{17}N_2O_6$	345.1082	345.1081	0.1	0.29
nl: - Caff/Glc/CO_2	$C_{16}H_{17}N_2O_4$	301.1186	301.1183	0.3	1.00
nl: - Caff /Glc/2CO_2/2H	$C_{15}H_{15}N_2O_2$	255.1121	255.1128	-0.7	-2.74
15-decarboxy-malabarin	$C_{32}H_{33}N_2O_{14}$	669.1932	669.1926	0.6	0.90
nl: - Caff	$C_{23}H_{27}N_2O_{11}$	507.1617	507.1609	0.8	1.58
nl: - Caff/Glc	$C_{17}H_{17}N_2O_6$	345.1083	345.1081	0.2	0.58
2,17-decarboxy-malabarin	$C_{31}H_{33}N_2O_{12}$	625.2026	625.2028	-0.2	-0.32
nl: - Caff	$C_{22}H_{27}N_2O_9$	463.1709	463.1711	-0.2	-0.43
nl: - Caff/Glc	$C_{16}H_{17}N_2O_4$	301.1181	301.1183	-0.2	-0.66
nl: - Caff/Glc/CO_2/2H	$C_{15}H_{15}N_2O_2$	255.1125	255.1128	-0.3	-1.18

[a] nl – neutral losses from $[M + H]^+$, Coum – coumaroyl, Caff – caffeoyl, Glc – glucosyl

Table S6. TEAC Values Determined for *B. alba* Extracts (B1 and B2), Gomphrenin, 6'-*O*-*E*-Coumaroyl-gomphrenin (Malabarin), 6'-*O*-*E*-4-Coumaroyl-gomphrenin (Globosin), 2-Decarboxy-gomphrenin, 17-Decarboxy-gomphrenin and 2,17-Bidecarboxy-gomphrenin as well as Caffeic Acid (Reference Compound) in the ABTS, FRAP and ORAC Tests.

No.	Sample name	IC_{50}		TEAC, mmol TROLOX/g DW					
		ABTS	±SD	ABTS	±SD	FRAP	±SD	ORAC	±SD
1	*B. alba* - extract B1	26.0[a]	±0.35	1.68[g]	±0.10	1.39[g]	±0.03	3.34[d]	±0.78
2	*B. alba* - extract B2	15.3[b]	±0.65	2.47[f]	±0.16	2.43[f]	±0.12	6.62[d]	±0.48
3	gomphrenin - Gp	3.65[e]	±0.31	9.87[b]	±0.15	10.8[a]	±0.40	15.9[c]	±1.5
4	malabarin - Caff-Gp	5.01[d]	±0.22	6.27[c]	±0.32	7.86[b]	±0.28	16.4[c]	±1.5
5	globosin - Coum-Gp	10.0[c]	±0.42	3.55[e]	±0.22	4.49[e]	±0.26	14.1[c]	±2.0
6	2-decarboxy-gomphrenin - 2-dGp	2.98[e]	±0.49	10.40[a]	±0.09	11.0[a]	±0.39	25.5[a]	±1.8
7	2,17-bidecarboxy-gomphrenin - 2,17-dGp	3.33[e]	±0.20	10.41[a]	±0.06	10.4[a]	±0.45	21.0[b]	±1.0
8	17- decarboxy-gomphrenin - 17-dGp	5.43[d]	±0.21	6.00[c,d]	±0.08	6.48[c]	±0.25	17.4[b,c]	±0.48
9	caffeic acid - Caff-acid	6.01[d]	±0.16	5.55[d]	±0.21	5.51[d]	±0.36	20.8[b]	±2.1

[a, b, c, d, e, f] - letter designation in the same column of statistically significant differences ($p \leq 0.05$) between samples according to Tuckey's test.
